# PUFAs, BDNF and lipoxin A4 inhibit chemical-induced cytotoxicity of RIN5F cells in vitro and streptozotocin-induced type 2 diabetes mellitus in vivo

**DOI:** 10.1186/s12944-019-1164-7

**Published:** 2019-12-10

**Authors:** Siresha Bathina, Undurti N. Das

**Affiliations:** 1 BioScience Research Centre and Department of Medicine, Gayatri Vidya Parishad Hospital, GVP College of Engineering Campus, Visakhapatnam, 530048 India; 2 Present Address: Department of Biotechnology, Gandhi Institute of Technology and Management (GITAM) Institute of Science, GITAM University, Visakhapatnam, Andhra Pradesh India; 3 UND Life Sciences, 2221, NW 5th St, Battle Ground, WA 98604 USA

**Keywords:** Brain derived neurotrophic factor, Polyunsaturated fatty acid, Lipoxin A4, RIN5F cells, Inflammation, Antioxidants

## Abstract

**Objective:**

To study whether minimal doses of arachidonic acid (AA), eicosapentaenoic acid (EPA) and docosahexaenoic acid (DHA) and lipoxin A4 (LXA4) and brain-derived neurotrophic factor (BDNF), when used in combination can protect RIN5F cells from chemical-induced cytotoxicity. As a corollary, to know whether plasma BDNF and LXA4 are altered in STZ-induced type 2 DM animals.

**Materials and methods:**

RIN5F cells, alloxan (AL), streptozotocin (STZ), doxorubicin (DB), and benzo(a)pyrene (BP) were used in this study. Chemical-induced apoptosis and changes in antioxidants, lipid peroxides and nitric oxide (NO) and LXA4 and BDNF levels in RIN5F cells were studied. Alterations in plasma concentrations of BDNF and LXA4 in STZ-induced type 2 diabetes animals was estimated.

**Results:**

BDNF, LXA4 and AA, EPA and DHA protected (*P* < 0.001 and *P* < 0.01 respectively) against AL/STZ/DB/BP-induced toxicity to RIN5F cells in vitro. AL/ STZ/DB/BP inhibited BDNF and LXA4 production by RIN5F cells and were restored to normal by AA, EPA and DHA. Sub-optimal doses of BDNF, LXA4, AA and EPA when used in combination protected against cytotoxic action of AL/STZ/DB/BP on RIN5F cells in vitro by restoring LXA4/BDNF levels and altered antioxidant/lipid peroxides/NO levels (*P* < 0.01) to normal. STZ (65 mg/kg)-induced type 2 diabetes mellitus animals showed reduced plasma BDNF and LXA4 levels (*P* < 0.001).

**Discussion:**

AL/STZ/DB/BP-induced cytotoxicity to RIN5F cells in vitro can be prevented by BDNF, LXA4 and AA. AL/STZ/DB/BP are cytotoxic, possibly, by suppressing the production of LXA4 and BDNF in RIN5F cells. STZ-induced type 2 DM animals have decreased plasma levels of LXA4 and BDNF.

**Conclusion:**

The results of the present study suggest that BDNF, LXA4, EPA, DHA, AA, GLA and BDNF protect pancreatic β cells from the cytotoxic action of various chemicals and prevent development of diabetes mellitus. LXA4 seems to be the mediator of these cytoprotective actions of BDNF and PUFAs suggesting a close interaction exists among these molecules (BDNF, PUFAs and LXA4). Hence, methods developed to deliver a combination of PUFAs (especially AA), LXA4 and BDNF may prevent development of diabetes mellitus (both type 1 and type 2).

## Highlights of the study

- BDNF, eicosapentaenoic acid (EPA) and docosahexaenoic acid (DHA), arachidonic acid (AA), gamma-linolenic acid (GLA) and lipoxin A4 (LXA4) protected RIN5F (rat pancreatic β) cells against alloxan (AL), streptozotocin (STZ), doxorubicin (DB) and benzo(a)pyrene (BP)-induced cytotoxicity in vitro.
- AL, STZ, DB, and BP inhibited BDNF and LXA4 (*P < 0.001*) production and altered antioxidant status of RIN5F cells in vitro which were restored to normal by EPA, DHA, AA and GLA.
- A combination of minimal doses of various PUFAs (10 μg/ml) and LXA4 and BDNF (50 ng/ml) showed synergistic action in protecting RIN5F cells against the cytotoxic action of AL, STZ, DB and BP.
- STZ (65 mg/kg)-induced type 2 diabetes mellitus animals showed a significant reduction in plasma BDNF (*P* < 0.001) and LXA4 (*P* < 0.001) levels*.*
- These results suggest that an interaction exists between BDNF and various PUFAs and AA, LXA4 and BDNF may function as endogenous cytoprotective and anti-diabetic molecules.

## Introduction

Both obesity and type 2 diabetes mellitus (type 2 DM) are assuming epidemic proportions in several countries that has been attributed to increased consumption of high fat diet or energy dense food and lack of exercise [1, 2]. Both passive and active smoking and exposure to environmental pollutants have also been linked to increasing incidence of type 2 DM [3–8]. Ability of insulin to stimulate glucose uptake is lower in cigarette smokers [9] and smoking increases abdominal fat distribution and a greater waist-to-hip ratio that can enhance insulin resistance [10]. Smoking increases free radical oxidative damage and oxidative stress [11]. Nicotine, carbon monoxide or other agents in tobacco smoke are likely to be toxic to pancreatic β cells and decrease insulin receptor sensitivity [12, 13], issues that can increase the risk of development of type 2 DM.

Benzo(a)pyrene (BP), one of the polycyclic aromatic hydrocarbons (PAHs), is an important component of coal tar, cigarette smoke, wood smoke, and burnt foods such as coffee and grilled foods [14]. BP and related compounds intercalate into DNA, interfering with transcription and enhance the risk of lung cancer. Intestinal cytochrome P450 subclass 1A1 (CYP1A1) protects the host from ingested carcinogens such as PAH including BP and its expression (CYP1 including CYP1A2 and CYP1B1) is dependent on a heterodimeric transcription factor consisting of the arylhydrocarbon receptor (AHR) and the AHR nuclear translocator (ARNT). The presence of ligands for TLR2 (toll-like receptor 2) of bacterial origin is crucial for detoxication of luminal carcinogens by CYP1A1 in the intestine. This indicates the complex interplay between the immune system of the host and intestinal bacteria with detoxication mechanisms [15]. More than 20% of the carbon in the universe is associated with PAHs, indicating that atmospheric pollutants such as BP and other PAHs have a role in the development of diabetes mellitus. Recent studies revealed that environmental chemicals such as bisphenol A (BPA), BP, and polychlorinated biphenyls (PCBs) induce generation of high levels of reactive oxygen species (ROS), a mechanism by which they may induce damage to islet cells and thus, produce diabetes mellitus [16]. BPA, BP and PCBs may also directly affect insulin secretion and alter the expression of key proteins involved in the cellular and endoplasmic reticulum stress response and thus, may contribute to the etiology of type 2 DM [17].

Doxorubicin (DB), a widely used anti-cancer drug, inhibits adipogenesis through the down-regulation of PPARγ and impairs blood glucose and lipid clearance that may aid in the development of hyperglycemia and hyperlipidemia that may result in glucotoxicity, lipotoxicity leading to inflammation and insulin resistance that are features of type 2 diabetes [18].

Brain-derived neurotrophic factor (BDNF) produced by several tissues in the body including brain, intestines, pancreas and adrenal gland behaves as a transmitter between immune and nervous systems and modulates inflammation [1, 19]. BDNF decreased food intake, increased energy expenditure and reversed hyperinsulinemia and ameliorated type 2 diabetes mellitus in experimental animals [1, 20, 21]. Plasma BDNF levels are reduced in subjects with obesity and type 2 DM [22, 23], though this has been disputed [24]. Previously, we showed that BDNF is not only a neurotrophic factor but also protects pancreatic β cells against the cytotoxic action of alloxan, streptozotocin (STZ), doxorubicin (DX) and BP in vitro and thus, may prevent diabetes mellitus [25]. We also reported that polyunsaturated fatty acids (PUFAs) such as linoleic acid (LA), γ-linolenic acid (GLA), dihomo-GLA (DGLA) and arachidonic acid (AA) of n-6 series and α-linolenic acid (ALA), eicosapentaenoic acid (EPA) and docosahexaenoic acid (DHA) of n-3 series and the anti-inflammatory metabolite of AA namely lipoxin A4 (LXA4) prevented cytotoxic action of alloxan against pancreatic β cells in vitro [26–30]. But it is not known whether BDNF and PUFAs (including LXA4) can interact and enhance each other’s action and thus, protect pancreatic β cells.

The results of the present study revealed that when minimal doses of BDNF and PUFAs and lipoxin A4 (LXA4) are supplemented together can prevent cytotoxic action of AL, STZ, BP and DB to rat pancreatic β cells (RIN5F) in vitro. In addition, BDNF and LXA4 enhanced the synthesis of each other by pancreatic β cells suggesting that PUFAs and BDNF interact and influence each other’s metabolism and action. Furthermore, plasma concentrations of BDNF and LXA4 were low in Wistar rats that developed STZ-induced type 2 diabetes implying that these molecules (BDNF and LXA4) have a role in the pathobiology of type 2 diabetes mellitus.

## Materials and methods

### Reagents

Alloxan (AL), streptozotocin (STZ) and benzo-(a)-pyrene (BP) were purchased from Sigma Aldrich (USA). Doxorubicin (DB) was purchased from Khandelwal Laboratories., (Thane, India). All culture media and additives were purchased from Sigma Aldrich Chemicals Pvt. Ltd., (Bangalore, India). BDNF and BDNF ELISA kits were purchased from Millipore, Massachusetts (USA), and lipoxin A4 (LXA4) ELISA kits were purchased from Oxford Biomedical Research (Michigan, USA).

### Cell culture conditions

An insulin-secreting rat insulinoma (RIN5F) cell line obtained from the National Center for Cell Science (Pune, India) was used in the present study. RIN5F cells were grown in RPMI1640 culture media (pH 7.4) supplemented with bicarbonate, 10 mM HEPES, 1 mM sodium pyruvate, 100 U/ml penicillin, 100 μg/ml streptomycin, 1.25 μg/ml amphotericin B, 10% fetal bovine serum (FBS) at 37 °C with 5% CO_2_. The cells were sub-cultured at regular intervals of 3–4 days when they are confluent. For cell culture studies, adherent cells were first washed with phosphate buffered saline (PBS, pH 7.4) and then treated with trypsin (0.25%) – EDTA (0.02%) for 3 min. Trypsin was immediately inactivated by addition of equal volume of FBS and centrifuged to pellet the cells which were used for various studies as described below.

### MTT studies

#### Dose and time optimization of AL/STZ/DB/BP on RIN5F

RIN5F were seeded at a density of 5 X 10^4^ cells /100 μl of culture media in 96-well plates. AL was dissolved in 50 mM of citrate buffer (pH 3.0) and STZ in 100 mM citrate buffer (pH 4.5). After 44 h of attachment period, cells were treated with different doses of AL (1 – 12 mM) for 1–3 h; STZ doses (1-30 mM) for 12–48 h and DB in saline at the concentrations of 6.25 ng/ml-800 ng/ml for 12- 48 h. At the end of each treatment period, cell viability was measured by MTT (3-(4,5-dimethylthiazole-2-yl)-2,5-diphenyltetrazolium bromide) assay. For studies with BP, 0.5 - 8 mM doses were tested at various time periods of 12–48 h and at the end of each treatment period MTT assay was performed in an ELISA plate reader (Multiskan EX, Thermo Scientific Ltd., USA). The cell growth percentage was expressed as percentage of cell growth compared with control in the same treatment group [25].

#### Dose and time optimization studies with BDNF on RIN5F cells

RIN5F cells seeded at a density of 5 X 10^4^ cells /100 μl of culture media in 96-well plates were exposed to different doses (1, 5, 10, 25, 50 and 100 ng/ml) of BDNF and incubated for various time periods (6, 12, 24, 48, 72 and 96 h) to test for its (BDNF) effect on the growth and viability of RIN cells by MTT assay as previously described [25].

#### Dose and time optimization studies with PUFAs on RIN5F cells

RIN5F cells were seeded at a density of 5 X 10^4^ cells/100 μl of culture media in 96-well plates. After 44 h of attachment period, cells were treated with various doses (5, 10, 15 μg/ml) of PUFAs: LA, AA, GLA, DGLA, ALA, EPA and DHA for 5 h. At the end of the treatment periods, cell viability was determined by MTT assay as described previously [25].

#### Dose and time optimization studies with LXA4 on RIN5F cells

RIN5F cells seeded at a density of 5 X 10^4^ cells /100 μl of culture media in 96-well plates were incubated with LXA4 (5, 10, 50 and 100 ng/ml for 6, 12, 24, 48, 72 and 96 h) at the end of which cell viability was determined by MTT assay as described previously [25].

#### Effect of BDNF, n-3 and n-6 PUFAs on AL/BP/DB/STZ induced cytotoxicity to RIN5F cells

This study was performed with 5 X 10^4^ cells /100 μl of culture media in 96-well plates. The RIN cells were treated with AL (6 mM)/STZ (20 mM)/DB (100 ng/ml)/BP (1.5 mM) and various doses of BDNF (10, 50 and 100 ng/ml) and PUFAs (15 μg/ml) depending on the experimental protocol to test their (BDNF and PUFAs) effect on chemical-induced (AL/STZ/DB/BP) cytotoxicity. Our preliminary studies revealed that maximum cytoprotective action of BDNF, LXA4 and various PUFAs against AL/BP-induced cytotoxicity to RIN5F cells was seen when the cells were pretreated with BDNF, LXA4, and PUFAs. On the other hand, BDNF, LXA4 and various PUFAs showed maximum cytoprotective action against STZ/DB when were added simultaneously with these cytotoxic chemicals. Hence, all studies were performed accordingly.

**Scheme 1:**
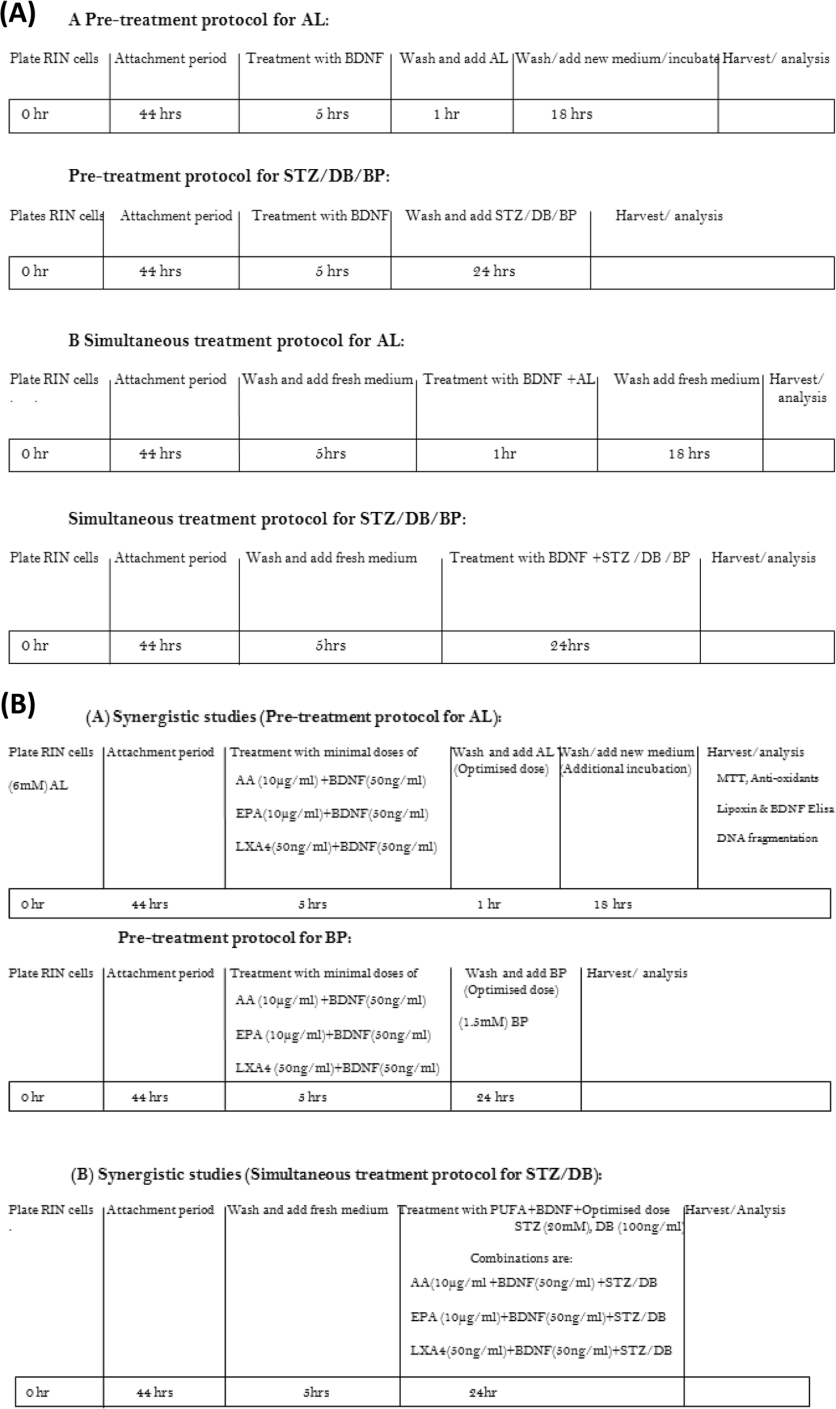
Protocols employed in the present study while testing the cytotoxic action of various chemicals (AL/BP/STZ/BP) and its modulation by PUFAs, BDNF and LXA4 on RIN5F cells in vitro.

Two types of treatment protocols (pre- and simultaneous) were employed in the present study. In the pre-treatment protocol, after 44 h of initial attachment period, RIN5F cells seeded in 96-well plate and incubated with different doses of BDNF (10, 50 and 100 ng/ml) and various PUFAs (15 μg/ml) for 5 h following which spent media was replaced with fresh media containing optimized AL (6 mM)/BP (1.5 mM) doses and incubated for an additional period of 1 h (AL) and 24 h (BP) respectively. At the end of this additional incubation period, cell viability was determined by MTT assay. In the simultaneous treatment protocol, RIN5F cells were exposed to BDNF/PUFAs and STZ/DB simultaneously and incubated for 24 h (before this addition, the cells were grown for 5 h in culture media). At the end of the treatment period, viability of cells was measured by MTT assay as described previously [25]. The protocol of these studies is given in Scheme 1. Our previous studies showed that PUFAs when used at 15 μg/ml showed optimal amount of cytoprotection against AL/STZ induced cytotoxicity to RIN5F cells in vitro [29, 30]. Hence, in the present study all PUFAs were used at 15 μg/ml for their possible cytoprotective action against AL/STZ/DB/BP induced cytotoxicity to RIN5F cells in vitro. In studies aimed at assessing possible synergism of the cytoprotective action of PUFAs and BDNF, 10 μg of AA/ml and EPA/ml and BDNF 50 ng/ml were used, which are less than the optimal doses (optimal dose of PUFAs: 15 μg/ml and BDNF: 100 ng/ml).

#### Effect of LXA4 on DB/BP induced cytotoxicity on RIN5F cells

Two types of experiments were carried out to study the effects of various doses of LXA4 on DB/BP-induced cytotoxicity as described below. In the pre-treatment protocol, RIN5F cells were first incubated with LXA4 for 5 h at the end of which the excess of LXA4 was removed and cell were incubated with optimized dose of BP. In the simultaneous treatment protocol, cells were incubated with medium for 5 h and then LXA4 and DB were added simultaneously and incubated for predetermined period. At the end of this incubation, cells were harvested and MTT assay [25] and other tests were performed. In all these studies, appropriate controls were used.

#### Study of effect of a combination of PUFAs + BDNF and BDNF + LXA4 on cytotoxicity induced by AL/BP (pre-treatment schedule) and STZ/DB (simultaneous treatment schedule) on RIN5F cells

These studies were performed with 5 X 10^4^ cells /100 μl of culture media in 96-well plates. RIN cells were treated with AL/STZ/DB/BP and sub-optimal doses of PUFAs (10 μg/ml): AA and EPA as representative of n-6 and n-3 series respectively and sub-optimal doses of BDNF/LXA4 (50 ng/ml) were used to test for their effect on chemical-induced cytotoxicity. All studies with AL/BP were performed using pre-treatment schedule whereas studies with DB/STZ were performed using simultaneous treatment schedule. Further details of these studies are described below.

After 44 h of initial attachment period, cells seeded in 96-well plate and incubated with different doses of BDNF (50 ng/ml) and PUFAs (10 μg/ml) for 5 h following which spent media was replaced with fresh media containing optimized AL dose (6 mM)/BP (1.5 mM) were incubated for an additional period of 1 h (AL) and 24 h (BP) respectively. At the end of this additional incubation period, cell viability was determined by MTT assay. In simultaneous treatment, RIN5F cells were exposed to BDNF (50 ng/ml) and PUFAs (10 μg/ml) and STZ/DB simultaneously and incubated for 24 h (before this addition, the cells were grown for 5 h in culture media). At the end of the treatment period, viability of the cells was measured by MTT assay as described previously [25]. The protocol for these two types of studies is given in Scheme 1.

#### Effect of a combination of LXA4 (50 ng/ml) and BDNF (50 ng/ml) on AL/STZ/DB/BP induced cytotoxicity to RIN5F cells

Our previous studies showed that LXA4 can protect RIN cells from the cytotoxic action of AL and STZ [27, 28] and pre-treatment schedule is more effective compared to simultaneous treatment schedule. Hence, in the present study experiments were performed only with DB and BP. In the previous studies [29, 30], we employed 1, 5, 10, 50 ng/ml of LXA4 to test its protective actions against the cytotoxic action of AL and STZ and noted that at 50 ng/ml LXA4 could restore ~ 70–80% viability of RIN cells. Hence, this 50 ng/ml of LXA4 is considered as the sub-optimal dose for synergistic studies in the present study.

After 44 h of initial attachment period, RIN cells were seeded in 96-well plates and incubated with LXA4 (50 ng/ml) and BDNF (50 ng/ml) individually and in combination (LXA4 + BDNF) depending on the treatment protocol for 5 h. At the end of this incubation period, spent media was replaced with fresh medium containing AL (6 mM)/BP (1.5 mM) and incubated for an additional 1 h (AL) and 24 h (BP) respectively. In the case of the study with AL, at the end of 1 h of incubation with AL, cells were incubated for an additional 18 h in fresh medium. At the end of the respective study periods, cell viability was determined by MTT assay [25]. To assess possible synergism between LXA4 and BDNF against the cytotoxic action of STZ/DB, these chemicals were added together to RIN cells as described above. When the individual and synergistic cytoprotective action of LXA4 (50 ng/ml) and BDNF (50 ng/ml) was tested against STZ/DB induced cytotoxicity against RIN5F cells, simultaneous protocol was employed wherein the incubation time with all chemicals was 24 h (before this addition, cells were grown for 5 h in culture medium). At the end of the treatment period, viable cell number was measured by MTT assay [25, 29, 30]. The details of this study are given in Scheme 1.

### Lipoxin A4 measurement

#### Effect of AL/STZ/DB/BP on LXA4 generation by RIN5F cells

RIN5F cells were plated at a density of 0.5 × 10^6^ cells/0.5 ml of culture media in 24-well plates. After 44 h of attachment period, cells were treated with AL/STZ/DB/BP as described below. At the end of the respective incubation periods, cells were treated with 100 μl of lysis buffer (20mMTris, 100 mM NaCl, 1 mM EDTA, 250 μl of 10% Triton X100). The lysate was collected and centrifuged at 10,000 rpm at 4 °C for 10 min to pellet the nuclei. The supernatant was used for measurement of LXA4 by ELISA kit per the manufacturer’s instructions (EA45, Oxford Biomedical Research, USA). The range of detection limit is 10-500 pg/ml and no cross reactivity with eicosanoids and sensitivity 10-15 pg/ml with intra and inter assay CVs of ±4.1%. To know the effect of AL/STZ/DB/BP on the ability of RIN cells to synthesize and secrete LXA4 the following doses of these chemicals were used: AL: 6 mM was added to RIN5F cells and incubated for 1 h; STZ: 20 mM was added to RIN5F cells and incubated for 24; DB: 100 ng/ml was added for 24; and BP: 1.5 mM was added for 24 h.

#### Effect of BDNF (50, 100 and 200 ng/ml) on LXA4 generation by RIN5F

RIN5F were seeded at a density of 5 X 10^4^ cells /100 μl of culture media in 96-well plates. Experiments were performed to study the effects of various doses of BDNF (50 and 100 ng/ml) and incubated for 24, 48 and 72 h on LXA4 generation by these cells. At the end of various incubation periods, cells were harvested and 100 μl of lysis buffer was added to each well and supernatants were analyzed for LXA4 by using LXA4 ELISA kit as mentioned above.

#### Effect of n-6 PUFAs (AA and GLA) and n-3 PUFAs (EPA and DHA) on LXA4 generation in the presence of AL/STZ/DB/BP by RIN5F cells

RIN5F cells were plated at a density of 0.5 × 10^6^ cells/0.5 ml of culture media in 24-well plates. After 44 h of attachment period, cells were supplemented with 15 μg/ml of AA, GLA, EPA and DHA to test for their ability to restore cytotoxic agents (AL/STZ/DB/BP)-induced decrease in LXA4 secretion. At the end of the respective incubation periods, the cells were harvested and 100 μl of lysis buffer was added to each well. The lysate was collected and centrifuged at 10,000 rpm at 4 °C for 10 min to pellet the nuclei. LXA4 was measured in the supernatants as described above.

#### Effect of AA and BDNF individually and in combination on LXA4 generation by RIN5F cells in the presence of AL/STZ/DB/BP

To study the effect of AL and BP, RIN5F cells were pretreated with 15 μg/ml of AA or BDNF (100 ng/ml) or in combination (BDNF+AA) for 5 h at the end of which spent media was replaced with fresh media containing optimized AL dose (6 mM)/BP (1.5 mM) and incubated for an additional 1 h in case of AL and 24 h with BP. At the end of this incubation, cells were harvested and lysed and LXA4 levels in the supernatants were measured according to instructions of ELISA kit. For studies with STZ and DB, 44 h after attachment period, RIN5F cells were treated with 15 μg/ml of AA with or without STZ (20 mM) or DB (100 ng/ml) for 24 h. At the end of the incubation, cells were harvested, lysed and LXA4 was measured in their supernatants by EA45 kit as described above.

#### Effect of BDNF (100 ng/ml) on LXA4 generation by AL/STZ/DB/BP-treated RIN5F cells

RIN5F cells were plated at a density of 0.5 × 10^6^ cells/0.5 ml of culture media in 24-well plates. After 44 h of attachment period, cells were pre-treated with optimal dose of 100 ng/ml of BDNF for 5 h in the pre-treatment schedule following which spent media was replaced with fresh media containing AL (6 mM)/BP(1.5 mM) and incubated for an additional 1 h with AL and 24 h with BP respectively. At the end of the respective incubation periods, cells were harvested, lysed and LXA4 levels in the supernatants were measured using LXA4 ELISA kit per the manufacturer’s instructions (EA45, Oxford Biomedical Research, USA). Similar study was performed with or without STZ (20 mM) / DB (100 ng/ml) wherein RIN5F cells were treated with 100 ng/ml of BDNF for 24 h at the end of which cells were harvested, lysed and measured for their content of LXA4 in their supernatant by EA45 kit.

#### Synergistic effect of AA (10 μg/ml) and BDNF (50 ng/ml) on LXA4 generation by AL/STZ/DB/BP-treated RIN5F cells

For this study, RIN5F cells seeded in 96-well plates, after 44 h of initial attachment period the cells were first incubated with AA (10 μg/ml) and BDNF (50 ng/ml) individually and in combination for 5 h after which the spent media was replaced with fresh media containing optimized AL dose (6 mM)/BP (1.5 mM) and incubated for an additional time of 1 h (AL) and 24 h (BP). At the end of this incubation period, cells were harvested, lysed and LXA4 levels in their supernatants were measured according to instruction of kit mentioned above. Similar study was also performed with STZ/DB wherein both STZ/DB and AA (10 μg/ml) and BDNF (50 ng/ml) were added individually (AA or BDNF) and in combination (AA + BDNF) and incubated for an additional 24 h (before this addition, cells were grown for 5 h). At the end of this incubation period, cells were harvested, lysed and LXA4 was measured in their supernatants by EA45 kit.

### BDNF measurement

#### Effect of optimized doses of AL/STZ/DB/BP on BDNF secretion by RIN5F cells

RIN5F cells were plated at a density of 0.5 × 10^6^ cells/0.5 ml of culture media in 24-well plates. After 44 h of attachment period, cells were treated with AL/STZ/DB/BP as described below. At the end of the various incubation periods, cells were treated with 100 μl of lysis buffer (20mMTris, 100 mM NaCl, 1 mM EDTA, 250 μl of 10% Triton X100). The lysate was collected and centrifuged at 10,000 rpm at 4 °C for 10 min to pellet the nuclei and the supernatant was used for measurement of BDNF by ELISA. For studies with AL: 6 mM was added to RIN5F cells and incubated for 1 h; while for studies with STZ: RIN5F cells were incubated with 20 mM for 24; for studies with DB: 100 ng/ml was added for 24; whereas for studies with BP: 1.5 mM was added for 24 h. The lysates of various treatments were collected and centrifuged at 10,000 rpm at 4 °C for 10 min to pellet the nuclei. The cell supernatant was used for measurement of BDNF by Elisa (CYT 306 kit) as per instructions of the manufacturer (EMD Millipore 290 Concord Road 01821 Billerica, MA, USA).

#### BDNF secretion by RIN5F cells in the presence of alloxan (2, 4 and 6 mM), STZ (20 and 40 mM), DB (100 and 200 ng/ml) and BP (1, 2 and 4 mM)

RIN5F cells were seeded at a density of 0.5 × 10^6^/well in 24-well culture plates in 500 μl of RPMI1640 and treated with different doses of alloxan (2, 4 and 6 mM), STZ (20 and 40 mM), DB (100 and 200 ng) and BP (1, 2 and 4 mM) for different periods of time. At the end of specific incubation periods, cells were trypsinized and centrifuged at 250 g for 10 min at room temperature. The cell pellet obtained was treated with 50 μl of lysis buffer (20mMTris + 100 mM NaCl+ 1 mM EDTA+ 250 μl of 10%Triton X) and the lysate was spun at 10,000 rpm at 2-4C for 10 min to pellet the nuclei. The cell supernatant was used for measurement of BDNF by Elisa (CYT 306 kit) as per instructions of the manufacturer (EMD Millipore 290 Concord Road 01821 Billerica, MA, USA). The range of detection limit is 7.8–500 pg/ml and no cross reactivity with eicosanoids and sensitivity 7.8 pg/ml with intra (+ 3.7%) and inter assay CVs of ±8.5%.

#### Effect of LXA4 (10, 25, 50 and 100 ng/ml) on the secretion of BDNF by RIN5F cells

RIN5F were seeded at a density of 5 X 10^4^ cells /100 μl of culture media in 96-well plates. The cells were treated with various doses of LXA4 (10, 25, 50 and 100 ng/ml) for different periods (12, 24 and 48 h). At the end of the incubation, cells were trypsinized, lysed and their supernatant was used for measuring BDNF as per instructions of CYT306 kit.

#### Effect of n-6 (AA and GLA) and n-3 PUFAs (EPA and DHA) on BDNF secretion by AL/STZ/DB/BP-treated RIN5F cells

RIN5F cells, seeded at a density of 0.5 × 10^6^ cells /0.5 ml of culture media in 24-well plates. After 44 h of attachment period, cells were treated with 15 μg/ml of AA, GLA, EPA and DHA following which cells were exposed to 1 h with AL and 24 h with BP. While testing for the effect of STZ and DB, RIN5F cells were incubated with PUFAs and STZ and DB simultaneously for 24 h (after 44 h of attachment period). At the end of the respective treatment periods, cells were harvested and lysed using the lysate buffer. The lysate was collected and centrifuged to pellet the nuclei. The cell supernatant was used for measurement of BDNF by using CYT306 kit.

#### Study of possible synergistic action of sub-optimal doses of AA and LXA4 on BDNF secretion by AL/STZ/DB/BP-treated RIN5F cells

After 44 h of attachment period, RIN5F cells seeded in 96-well plates, were incubated with sub-optimal doses of AA (10 μg/ml) and LXA4 (50 ng/ml) individually and in combination (AA + LXA4) for 5 h. At the end of this incubation period, spent media was replaced with fresh media containing optimized doses of AL (6 mM)/ BP (1.5 mM) and incubated for an additional 1 h (AL) and 24 h (BP). When the experiment was performed to study the effect of STZ/DB, RIN5F cells were incubated with AA ± LXA4 and STZ/DB simultaneously for 24 h. At the end of the respective incubation periods, cells were harvested, lysed and centrifuged and the resultant supernatants obtained were assessed for their BDNF content as per the instructions of the kit as described above.

### DNA fragmentation studies

#### Study of possible synergistic action of AA and BDNF against AL/STZ-induced apoptosis of RIN5F cells

RIN5F cells, plated at a density of 1 X 10^6^ cells/ml in 24-well plates, were pre-treated with AA (10 μg/ml) and BDNF (50 ng/ml) for 5 h followed by AL (6 mM) for an additional 1 h. On the other hand, for studies with STZ (20 mM), cells were exposed to STZ and AA + BDNF simultaneously for 24 h. At the end of the respective treatment periods, spent media was removed, cells were washed with PBS (pH 7.4) and trypsinized. Cells were centrifuged at 250 g for 10 min at room temperature and the cell pellet was lysed using 0.5 ml of lysis buffer (10mMTris + 75 mM NaCl+ 2 mM EDTA). Later DNA was extracted from the lysate by phenol-chloroform method and loaded (1μg) on 1.5% gel and run at 50 V for 2 h. This study was performed only with AL/STZ as representative of the cytotoxic agents (AL/STZ/BP/DB) and studied for the effect of a combination of sub-optimal doses of AA and BDNF as our previous studies revealed that optimal doses of BDNF and AA can prevent apoptosis induced by AL/STZ/BP/DB [26, 29, 30].

#### Estimation of various antioxidants in RIN5F cells

RIN5F cells, seeded at a density of 5 X 10^5^ cells/ml of culture media in 24-well plates were treated with optimized doses of AL/STZ/DB/BP after 44 h of attachment period. For studies with AL/BP, cells were pre-treated with these chemicals for 1 h and 24 h respectively following which the cells were exposed to AA (10 μg/ml) and BDNF (50 ng/ml) and AA (10 μg/ml) + BDNF 50 ng/ml for an additional 5 h. For studies with STZ/DB, RIN5F cells were incubated with these chemicals and AA ± BDNF simultaneously for 24 h. At the end of respective treatment periods, spent media was collected and cells were washed with PBS (pH 7.4) following which cells were lysed with lysis buffer (20 mM Tris, 100 mM NaCl, 1 mM EDTA& 0.5% of 10% Triton-X) and the lysates were used for the measurement of various antioxidant enzymes. In this study, the following antioxidant enzymes were measured as described previously: catalase, superoxide dismutase, glutathione-S-transferase and glutathione peroxidase [24, 27, 28]. Both spent media and cell lysate were used for estimation of lipid peroxides and nitric oxide by Griess reagent [26, 29, 30].

##### In vivo study

To verify whether the in vitro results are relevant to an in vivo situation, we measured plasma LXA4 and BDNF levels in STZ-induced type 2 diabetes mellitus in Wistar rats which were 3 to 4wk old, purchased from National Institute of Nutrition, (Hyderabad, India). The animals were housed at 25^0^ C room temperature with 12-h dark and 12-h light cycle. Animals weighing around 180 g were segregated into two groups of 10 animals: controls received PBS and citrate buffer; diabetic group received STZ (65 mg/kg) dissolved in citrate buffer. Experiment was approved by Institutional Animal Ethical Committee.

##### Induction of type 2 diabetes mellitus

Type 2 DM was induced in Wistar male rats by using nicotinamide (NAD) and STZ as previously described [30]. Freshly prepared 175 mg/kg body weight nicotinamide in PBS was administered intraperitoneally. After 15mins the animals received freshly prepared 65 mg/kg body weight STZ in 50 mM citrated buffer pH 4.5 intraperitoneally as described previously (Fig. 9a, [28]). Fasting blood glucose was measured by using Accu-Check blood glucose meter on 10th, 20th and 30th day from day 1 of the injection of STZ.

#### Estimation of blood glucose, food intake and body weight

The animals were confirmed to have developed diabetes when fasting blood glucose levels were > 250 mg/dL measured using Accucheck glucometer. Body weight and food consumption was measured twice a week. The total duration of the study was 30 days from the day of the injection of STZ (Fig. 9a). At the end of 30 days, animals were sacrificed to collect blood and various tissues for further studies. All samples were stored at − 80^0^ C till further analysis.

##### BDNF Elisa

BDNF was measured in the plasma samples of STZ and control samples by ELISA according to manufacturer instructions (CYT306).

##### Estimation of plasma lipoxin A4

LXA4 was measured in the plasma samples of the test groups by ELISA (Oxford Biomedical Research Company, MI, USA) as per the manufacturer’s instructions (EA45).

#### Statistical analysis

All studies were repeated thrice each time in triplicate. All results are expressed as mean ± SEM and all values obtained were analyzed employing paired t-test with equal variance in Microsoft Excel statistical analysis tool. All values are presented as mean ± SEM.

## Results

### MTT studies

#### Effect of AL on viability of RIN5F cells

**Fig. 1:**
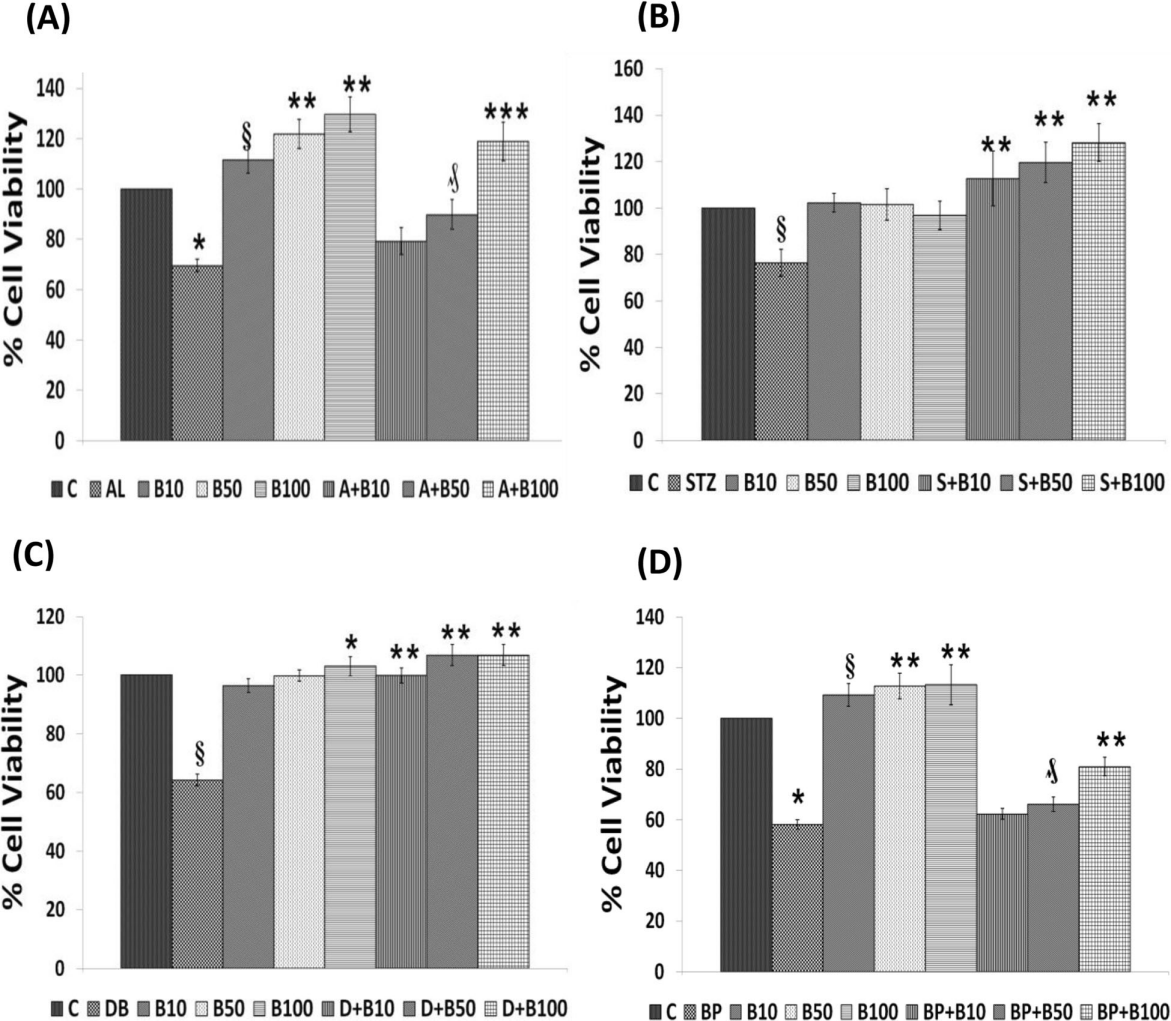
Effect of BDNF (10, 50 and 100 ng/ml) pre-treatment (in case of AL/BP) and simultaneous-treatment (in case of STZ/DB) induced cytotoxicity to RIN5F cells in vitro. **a**) Effect of Pre-treatment with BDNF on AL (6 mM)-induced toxicity to RIN5F cells. All values expressed as mean ± SEM. § *P* < 0.001 vs untreated control, ***P < 0.001 ₰ *P* < 0.01 vs alloxan-treated group. **P < 0.01 vs respective untreated control. (B = Brain derived neurotrophic factor). **b**) Effect of Simultaneous -treatment with BDNF on STZ (20 mM)-induced toxicity to RIN5F cells. All values expressed as mean ± SEM. **c**) Effect of Simultaneous- treatment with BDNF on doxorubicin (100 ng/ml) induced toxicity to RIN5F cells. All values expressed as mean ± SEM. § *P* < 0.001 vs untreated control, **P < 0.001 vs doxorubicin-treated group. *P < 0.01 vs respective untreated control. **d**) Effect of Pre-treatment with BDNF on BP (1.5 mM) - induced toxicity to RIN5F cells. All values expressed as mean ± SEM. *P < 0.001 vs untreated control, *# P* < 0.05; ₰ *P* ≤ 0.001 vs BP-treated group. **P ≤ 0.05 § P < 0.01 vs respective untreated control group. (B = Brain derived neurotrophic factor). All the above set of experiments were done in triplicate on three separate occasions (*n* = 9) and all values expressed as mean ± SEM

AL selectively inhibits glucokinase, the glucose sensor of beta cell and inhibits glucose-stimulated insulin secretion that leads to a state of insulin-dependent diabetes (type 1 DM) by its ability to induce ROS formation. In a previous study, it was found that when RIN5F cells were exposed to AL (1–12 mM) for different periods of time (1–3 h), AL showed a significant (*P < 0.001*) and dose-dependent cytotoxic action on the viability of RIN5F cells (data not shown, 27). Based on these results (23, Fig. 1a), we used in the present study a dose of 6 mM AL and exposure time of 1 h, conditions under which ~ 53% of the cells were found to be viable (see Additional file 1: Figure S1).

#### Effect of STZ on viability of RIN5F cells

STZ enters pancreatic beta cells by GLUT2 receptor and causes alkylation of DNA. In a previous study, we observed that RIN5F cells when exposed to different concentrations of STZ (1–30 mM) and incubated for 12–48 h produced a significant (*P < 0.001*) and dose-dependent decrease in the proliferation of RIN5F cells (data not shown, 30). STZ when used at 20 mM for 24 h, reduced viability of RIN5F cells ~ 50% and hence, all studies in the present investigation were performed using this protocol (Fig. 1b, 30) (see Additional file 1: Figure S1).

#### Effect of DB on viability of RIN5F cells

DB, an anthracycline compound that works by intercalating with DNA, generates free radicals and cause peroxidation of plasma membrane. Previously, we showed a dose-dependent cytotoxicity (*P < 0.001*) of DB on the viability of RIN5F cells when tested at 6.25-800 ng/ml and incubated for 12, 24 and 48 h (data not shown, 25). Based on these results [25], the present investigation was performed using 100 ng/ml of DB and an incubation time of 24 h which gave ~ 57% viability of RI5F cells (Fig, 1C, 25) (see Additional file 1: Figure S1).

#### Effect of BP on the viability of RIN5F cells

BP, an environmental pollutant, is a carcinogen and its reactive metabolites bind to cellular protein and nucleic acids forming DNA adducts. In a previous study, we noted that RIN5F cells when exposed to BP from 0.5 to 8 mM for 12, 24 and 48 h (data not shown, 25), there was a significant (*P < 0.001*) and a dose dependent decrease in the viability of cells [25]. Based on these results, in the present investigation we employed 1.5 mM of BP and an incubation time of 24 h (Fig. 1d, 25) (see Additional file 1: Figure S1).

#### Effect of BDNF on the viability of RIN5F cells

Previously, we showed that when RN5F cells were exposed to various doses of BDNF (1, 5, 10, 25, 50 and100 ng/ml) and incubated for different periods (1, 6, 12 and 24 h) it is not toxic at all the doses and incubation periods tested (data not shown, 23) (see Additional file 2: Figure S2). In fact, it was observed that 1, 25 and 50 ng/ml of BDNF enhanced the viability of RIN5F cells at the end of 6 h of incubation (data not shown, 23). Based on these results [25], we used 10, 50 and 100 ng/ml doses of BDNF in the present investigation.

#### Effect of n-3 and n-6 PUFAs (15 μg/ml) on RIN5F cells

Our previous study indicated that various PUFAs when added at 5, 10, 15 μg/ml (LA, AA, GLA, DGLA, ALA, EPA and DHA) to RIN5F cells and incubated for various periods of time (6, 12, 24, 48, 72 and 96 h), they (PUFAs) do not have any significant adverse effects on the viability of RIN5F cells in vitro (data not shown, 29, 30) (see Additional file 3: Figure 3).

#### Effect of LXA4 on viability of RIN5F cells

In a similar fashion, effect of various doses of LXA4 (10, 25, 50 and 100 ng/ml) on the viability of RIN5F cells was also tested at different periods of incubation (6, 12, 24,48,72 and 96 h). The results of this study revealed that at all the concentrations tested LXA4 does not have any significant action on their viability (data not shown, 29, 30) (see Additional file 3: Figure S3). In fact, it was observed that LXA4 enhanced the viability of (possibly by enhancing their proliferation) of RIN5F cells when incubated with 25, 50 and 100 ng/ml. Based on these results, we performed the present study with 10, 25, 50 and 100 ng/ml doses of LXA4.

#### Effect BDNF on AL/STZ/DB/BP-induced cytotoxicity to RIN5F cells

Based on the various preliminary results obtained with AL/STZ/DB/BP and BDNF/LXA4, in the present study we tested the effect of 10, 50 and 100 ng/ml of BDNF against the cytotoxic action of AL (6 mM) and BP (1.5 mM) using pre-treatment protocol and STZ (20 mM) and DB (100 ng/ml) using simultaneous treatment schedule protocol (see Scheme 1). These results shown in Fig. 1a-d revealed that 50 ng/ml and 100 ng/ml of BDNF have a significant cytoprotective action (**P < 0.001, **P < 0.01 and ₰P ≤ 0.05). against the cytotoxic action of AL, STZ, DB and BP against RIN5F cells respectively in vitro.

#### Effect of n-3 and n-6 PUFAs on AL/STZ/DB/BP-induced growth inhibition of RIN5F cells

**Fig. 2:**
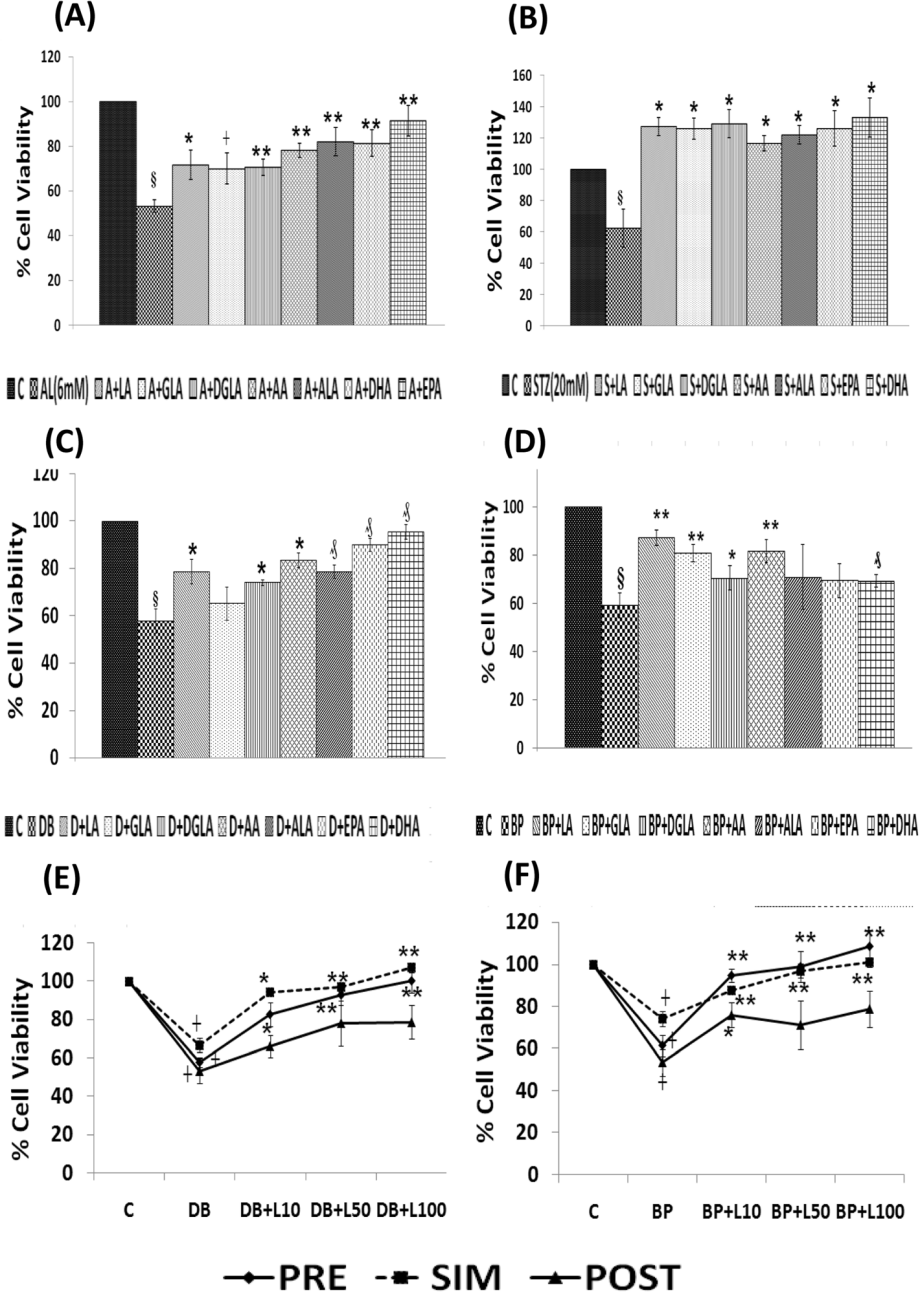
Effect of various n-3 and n-6 PUFAs treatment (pre- in case of AL/BP and simultaneous treatment in case of STZ/DB on chemical-induced cytotoxicity to RIN cells in vitro*.* **a** Effect of pre-treatment with different PUFAs (15 μg/ml) on AL (6 mM)-induced toxicity to RIN cells. All values expressed as mean ± SEM. § *P* < 0.001 vs untreated control, ┼ *P* ≤ 0.05, *P* < 0.02, **P < 0.001 vs alloxan-treated group. **b** Effect of simultaneous-treatment with different PUFAs (15 μg/ml) on STZ (20 mM)-induced toxicity to RIN cells. All values expressed as mean ± SEM. § *P* < 0.001 vs untreated control, *P < 0.001 vs STZ-treated group. **c** Effect of simultaneous-treatment with different PUFAs (15 μg/ml) on DB (100 ng/ml)-induced toxicity to RIN cells. All values expressed as mean ± SEM. § *P* < 0.001 vs untreated control, *P < 0.001; ₰*P* ≤ 0.01 vs DB-treated group. **d** Effect of pre-treatment with different PUFAs (15 μg/ml) on BP (1.5 mM)-induced toxicity to RIN cells. All values expressed as mean ± SEM. § *P* < 0.001 vs untreated control, ┼ *P* ≤ 0.05, *P < 0.02, ₰ *P* ≤ 0.01, **P < 0.001 vs BP treated group (LA, linoleic acid; GLA, γ-linolenic acid; DGLA, dihomo- γ-linolenic acid; AA, arachidonic acid; ALA, α-linolenic acid; EPA, eicosapentaenoic acid; DHA, docosahexaenoic acid). **e** Effect of simultaneous -treatment with lipoxin A4 (L) on DB (100 ng/ml)-induced toxicity to RIN cells. All values expressed as mean ± SEM. ┼ *P* ≤ 0.001 vs untreated control, *P < 0.01; **P < 0.001 vs DB treated group. **f** Effect of pre -treatment with lipoxin A4 (L) on BP (1.5 mM)-induced toxicity to RIN cells. All values expressed as mean ± SEM. ┼ *P* ≤ 0.001 vs untreated control, **P < 0.001 vs BP treated group. All the above set of experiments were done in triplicate on three separate occasions (*n* = 9) and all values expressed as mean ± SEM

To evaluate the effect of various PUFAs on AL/STZ/DB/BP-induced growth inhibitory action, RIN5F cells were treated with 15 μg/ml of PUFAs (LA, AA, GLA, DGLA, ALA, EPA and DHA) and their possible cytoprotective action was tested against AL/BP using pre-treatment schedule and against STZ/DB using simultaneous treatment schedule as described above under materials and methods section and as shown in scheme 1. It is evident from the results shown in Fig. 2a that AL-induced growth inhibition of RIN5F cells was prevented by all the PUFAs tested (┼ P ≤ 0.05, *P < 0.02, ** P < 0.001). Of all the PUFAs tested, AA, EPA and DHA are the most effective cytoprotective agents compared to other PUFAs (*p* < 0.001). On the other hand, growth inhibition induced by BP was prevented to a significant degree by LA, GLA and AA, whereas other PUFAs were not that effective (Fig. 2d).

It is evident from the results shown in Fig. 2b and c that when RIN5F cells were simultaneously treated with STZ and PUFAs, all PUFAs were almost equally effective in preventing cytotoxic action of STZ against RIN5F cells (Fig. 2b). On the other hand, growth inhibition induced by BP was prevented to a significant degree by LA, GLA and AA, whereas other PUFAs were not that effective (Fig. 2d). Of all, LA, DGLA, AA, ALA, EPA and DHA were the most effective fatty acids in preventing the cytotoxic action of DB (Fig. 2c). It may be mentioned here that our previous studies showed that both COX-2 (cyclo-oxygenase-2) and lipoxygenase (LOX) inhibitors did not block the cytoprotective actions of AA and other PUFAs [29, 30] suggesting that prostaglandins (PGs), thromboxanes (TXs) and leukotrienes (LTs) are unlikely to have any significant role in their (PUFAs) cytoprotective action. Hence, results with COX and LOX inhibitors are not shown here. In contrast to this, previously we observed that both alloxan and STZ-induced cytotoxic action on RIN5F cells can be prevented by LXA4, an anti-inflammatory metabolite of AA. Hence, in the present study further studies were performed with LXA4 to know whether LXA4 is affective against the cytotoxic action of DB and BP.

#### Effect LXA4 on DB/BP-induced cytotoxicity to RIN5F cells in vitro

The results of the study with DB (100 ng/ml) using the simultaneous treatment protocol given in Fig. 2e showed that of the three doses tested (10, 50 and 100 ng/ml), 50 ng and 100 ng of LXA4 can protect the cells against DB-induced growth inhibition. Similarly, BP (1.5 mM)-induced growth inhibition of RIN5F cells using pre-treatment protocol was also prevented by 100 ng/ml of LXA4 (Fig. 2f). In Fig. 2f, we are showing the results obtained with DB and BP though we performed similar studies with AL and STZ. Previously, we observed that even AL and STZ-induced growth inhibition of RIN5F cells can be prevented by LXA4 (data not shown, 29 and 30). Hence, in the present report we are showing results obtained with DB and BP only as examples of simultaneous and pre-treatment schedules respectively. Since 100 ng/ml of LXA4 gave optimal results, we performed synergistic studies using LXA4 50 ng/ml as the sub-optimal dose in these studies. Similarly, our previous studies [25] showed that 100 ng/ml of BDNF showed optimal protection against AL/STZ/BP/DB-induced apoptosis of RIN5F cells. Hence, synergistic studies with BDNF were performed using 50 ng/ml of BDNF that is considered as suboptimal dose of this molecule (BDNF).

#### Studies with a combination of sub-optimal doses of AA/EPA, and LXA4 and BDNF against cytotoxic action of various chemicals on RIN5F cells in vitro

Since PUFAs, LXA4 and BDNF have cytoprotective action against AL/STZ/BP/DB-induced cytotoxicity on RIN5F cells in vitro, we next studied whether a combination of suboptimal doses of PUFAs (10 μg/ml), LXA4 (50 ng/ml) and BDNF (50 ng/ml) will show significant protective action. This study was performed using pre-treatment protocol with AL/BP and simultaneous protocol with STZ/DB. In this study, we employed AA and EPA as representative of n-6 and n-3 PUFAs respectively.

#### Effect of a combination of sub-optimal doses of AA (10 μg/ml) and BDNF (50 ng/ml) against cytotoxic action of AL/BP/STZ/DB on RIN5F cells

**Fig. 3:**
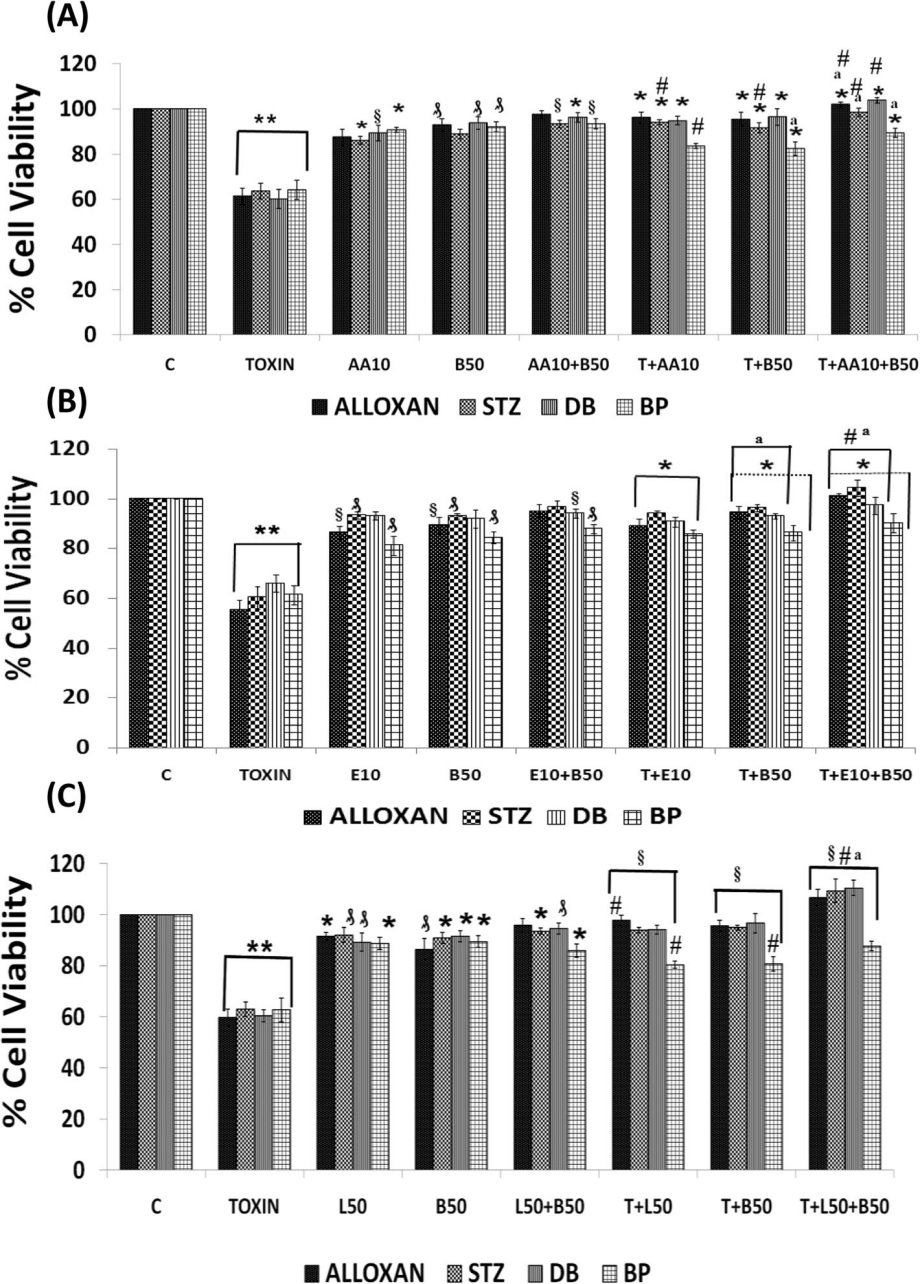
Synergistic effect of arachidonic acid (AA), eicosapentaenoic acid (E), lipoxin A4 (L) and BDNF (B) on chemical-induced cytotoxicity (AL/STZ/DB/BP) to RIN cells in vitro. **a**) Synergistic effect of AA and BDNF on AL/STZ/DB/BP-induced cytotoxicity to RIN5F cells in vitro., **P ≤ 0.001; ₰ *P* ≤ 0.01 § *P* < 0.05 vs untreated control; *P < 0.001 respective treated group vs toxin (T) (i.e., T vs T + AA10; T vs T + B50; T vs T + AA10 + B50); #*P* ≤ 0.01 vs individual and respective treated group (i.e., AA10 vs T + AA10; B50 vs T + B50; AA10 + B50 vs T + AA10 + B50) and ^a^*P* ≤ 0.05 vs treated and synergistic combination group (i.e., T + AA10 vs T + AA10 + B50; T + B50 vs T + AA10 + B50). **b**) Synergistic effect of eicosapentaenoic acid (E) and BDNF on AL/STZ/DB/BP-induced cytotoxicity to RIN5F cells in vitro, **P ≤ 0.001; ₰*P* ≤ 0.01 § *P* ≤ 0.05 vs untreated control; *P < 0.001 vs respective toxin treated group; ^a^*P* ≤ 0.01 vs respective individual treated group (i.e., E10 Vs T + E10; B50 Vs T + B50; E10 + B50 vs T + E10 + B50) and ^#^*P* ≤ 0.05 vs treated and synergistic combination group (T + B50 Vs T + E10 + B50). **c**) Synergistic effect of lipoxinA4 (L) and BDNF (B) on AL/STZ/DB/BP-induced cytotoxicity to RIN5F cells in vitro., **P ≤ 0.001; ₰*P* ≤ 0.05;*P ≤ 0.01 vs untreated control; §*P* < 0.001 vs respective toxin treated group; ^#^*P* ≤ 0.01 vs respective individual treated group (i.e. L50 vs T + L50; B50 vs T + B50; L50 + B50 vs T + L50 + B50) and ^a^*P* ≤ 0.05 vs treated and synergistic combination group (T + L50 vs T + L50 + B50; T + B50 vs T + L50 + B50). All the above set of experiments were done in triplicate on three separate occasions (*n* = 9) and all values expressed as mean ± SEM

In this study, RIN5F cells were treated with sub-optimal doses of AA and BDNF separately and in combination to know their effect against AL/STZ/DB/BP-induced growth inhibition. The results of this study given in Fig. 3a revealed that a combination of sub-optimal doses of AA and BDNF can effectively inhibit cytotoxic action of AL/STZ/BP/DB (pre-treatment protocol with AL/BP and simultaneous protocol with STZ/DB was employed in these studies) (*P < 0.001) compared to sub-optimal individual treatments with AA and BDNF (#*P ≤ 0.01*).

#### Effect of a combination of sub-optimal doses of EPA and BDNF against cytotoxic action induced by AL/BP/STZ/DB

Next, we evaluated possible synergistic action of EPA (E, 10 μg/ml) and BDNF (B, 50 ng/ml) against AL/BP-induced cytotoxic action on RINN5F cells using pre-treatment protocol and DB/BP using simultaneous treatment protocol. The results of this study shown in Fig. 3b revealed that a combination of sub-optimal doses of EPA and BDNF offered significant cytoprotection (*P < 0.001) to RIN5F cells when compared to AL/STZ/DB/BP alone treated groups (^*a*^*P ≤ 0.01*). The results given in Fig. 3b revealed that both EPA and BDNF even at sub-optimal doses can prevent the cytotoxic action of AL/STZ/DB against RIN5F cells (^*#*^*P ≤ 0.05*) though a combination of sub-optimal doses of EPA and BDNF is much more effective compared to individual doses of EPA and BDNF.

#### Synergistic studies with a combination of sub-optimal doses of LXA4 (50 ng/ml) and BDNF (50 ng/ml) against cytotoxic action of AL/STZ/DB/BP on RIN5F cells

We next evaluated possible synergistic action of a combination of sub-optimal doses of LXA4 (L, 50 ng/ml) and BDNF (B, 50 ng/ml) against the cytotoxic action of AL/STZ/DB/BP on RIN5F cells in vitro. This study was like the studies performed with AA and EPA except that here in the place of PUFAs (AA and EPA) LXA4 was used. LXA4 + BDNF showed significant *(§P < 0.001*) cytoprotection against AL/STZ/DB/BP-induced growth inhibition (Fig. 3c) when compared to respective individual cytotoxic agents *(*^*#*^*P ≤ 0.01)*. These results (Fig. 3c) suggest that a combination of suboptimal doses of LXA4 and BDNF, when used tested against the cytotoxic action of AL/STZ/DB/BP, showed cytoprotective action and produced a significantly increase in cell viability compared to results obtained with LXA4 (50 ng/ml) and BDNF (50 ng/ml) when used separately.

Of all the three synergistic combinations studied (i.e. AA+BDNF, EPA + BDNF and LXA4 + BDNF), the cytoprotective action shown by LXA4 + BDNF was much higher against the cytotoxic action of AL/STZ/DB/BP on RIN5F cells.

#### AL/STZ/DB/BP suppress LXA4 secretion by RIN5F cells in vitro

Since both AA and LXA4 could prevent the cytotoxic action of AL/STZ (data not shown) and DB/BP and the cytoprotective action of AA was not blocked by both COX and LOX inhibitors (data not shown), we next studied whether these chemicals alter the formation and secretion of LXA4 by RIN5F cells in vitro*.*

Pre–treatment schedule was employed to test the effect of AL/BP and simultaneous treatment schedule was used to test the effect of DB/STZ on LXA4 secretion by RIN5F cells in vitro. Results of this study given in Table 1 clearly showed that under the conditions employed, AL/STZ/DB/BP significantly reduced (*P < 0.001) secretion of LXA4 by RIN5F cells in vitro compared to untreated control.

**Table 1:** Summary of analysis of LXA4 and BDNF levels in the supernatant of RIN5F treated with Alloxan (Pre-treatment)/STZ (Simultaneous treatment) and sub-optimal doses of AA (10 μg/ml) and BDNF (50 ng/ml)

SUMMARY OF LIPOXIN A4 LEVELS AND BDNF LEVELS IN CHEMICAL(AL/STZ/DB/BP) INDUCED CYTOTOXICITY IN RIN5F CELLS INVITRO (supernatants)					
ELISA	GROUP	AL studiez (6 mM)	STZ studies (20 mM)	DB studies (100 ng/ml)	BP studies (1.5 mM)
LXA4 levels (ng/ml)	CONTROL	0.83 ± 0.2	0.91 ± 0.3	1.22 ± 0.3	0.75 ± 0.2
	CYTOTOXIN	0.5 ± 0.12*	0.37 ± 0.2*	0.67 ± 0.6*	0.41 ± 0.1*
BDNF levels (pg/ml)	CONTROL	36.9 ± 0.9	25.3 ± 0.8	37.3 ± 0.40	42.2 ± 0.3
	CYTOTOXIN	14.4 ± 0.2*	18.2 ± 0.3*	23.4 ± 0.36*	28.3 ± 0.9*

* P < 0.01

#### Effect of BDNF (10, 50 and 100 ng/ml) on LXA4 secretion by RIN5F cells in vitro

**Fig. 4:**
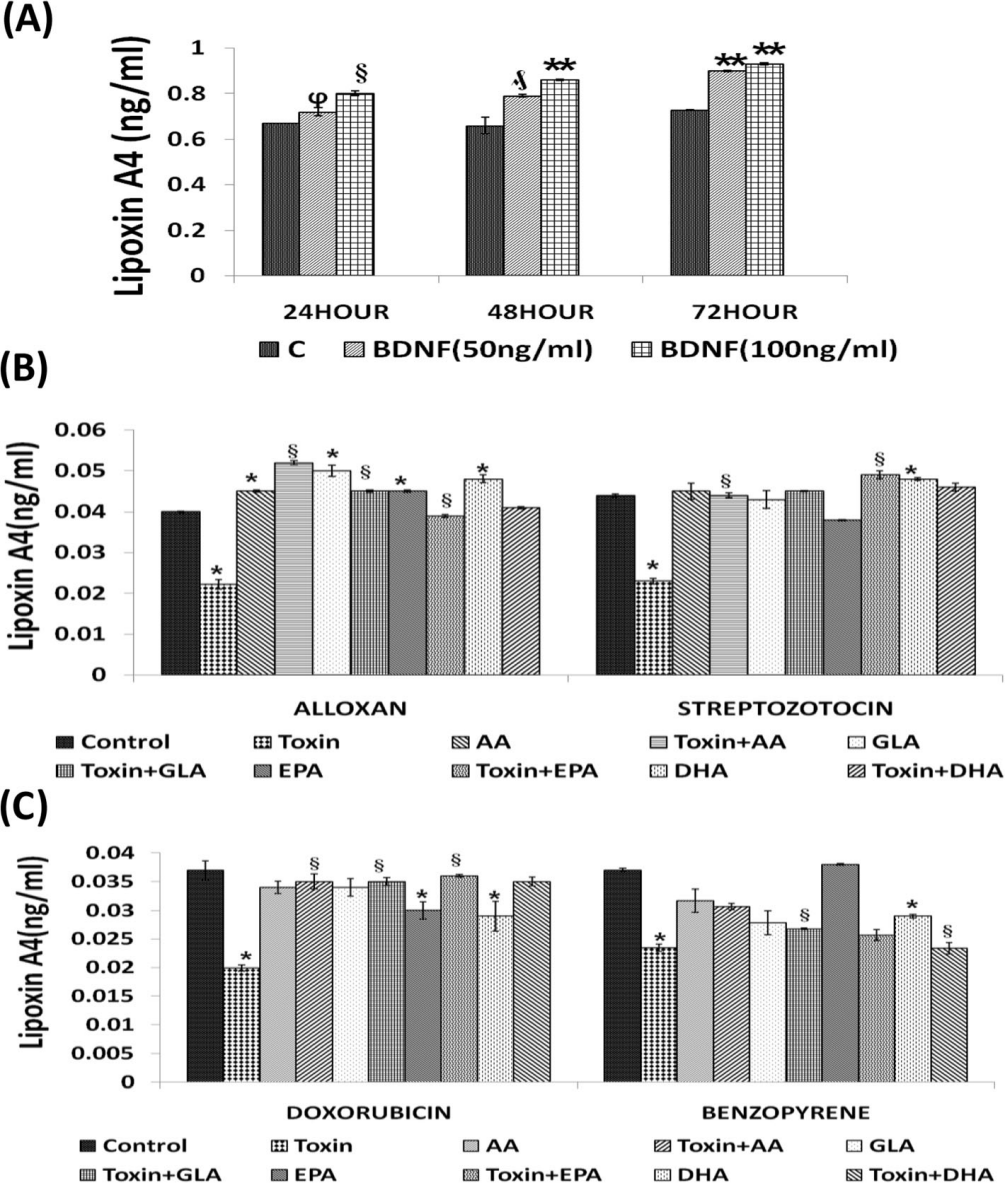
Measurement of lipoxin A4 in RIN5F cell supernatants. **a**) Effect of BDNF (50 and 100 ng/ml) on the secretion of LXA4 by RIN5F cells in vitro at the end of 24, 48 and 72 h of incubation. All values expressed as mean **±** SEM. ϕ *P* < 0.001, § *P* < 0.05, ₰ *P* < 0.01, **P < 0.001 Vs untreated control. BDNF-Brain derived neurotrophic factor. **b**) Effect of various PUFAs on the secretion of LXA4 by RIN5F cells in vitro that was suppressed by alloxan (6 mM) and STZ (20 mM). All values expressed as mean ± SEM. *P < 0.01 vs untreated control § *P* < 0.001 and vs toxin-treated group. AA, arachidonic acid; GLA, γ-linolenic acid; EPA, eicosapentaenoic acid; DHA, docosahexaenoic acid. **c**) Effect of various PUFAs on the secretion of LXA4 by RIN5F cells in vitro that was suppressed by BP (1.5 mM) and DB (100 ng/ml). All values expressed as mean ± SEM. *P < 0.01 vs Untreated control § *P* < 0.001 and vs toxin -treated group. AA, arachidonic acid; GLA, γ-linolenic acid; EPA, eicosapentaenoic acid; DHA, docosahexaenoic acid. All the above set of experiments were done in triplicate (*n* = 3) and all values expressed as mean ± SEM.

Since both BDNF and LXA4 can protect RIN5F cells against the cytotoxic action of AL/STZ/DB/BP, we next investigated whether BDNF enhances LXA4 formation and secretion by these cells in vitro. Interestingly we found that (Fig. 4a) there was a significant dose and time dependent increase (ϕP < 0.001, §P < 0.05, ₰P < 0.01, **P < 0.001) in the production and secretion of LXA4 by BDNF when compared to the untreated control.

#### Effect of PUFAs (15 μg/ml) on LXA4 secretion by AL/STZ/DB/BP-treated RIN5F cells

**Fig. 5:**
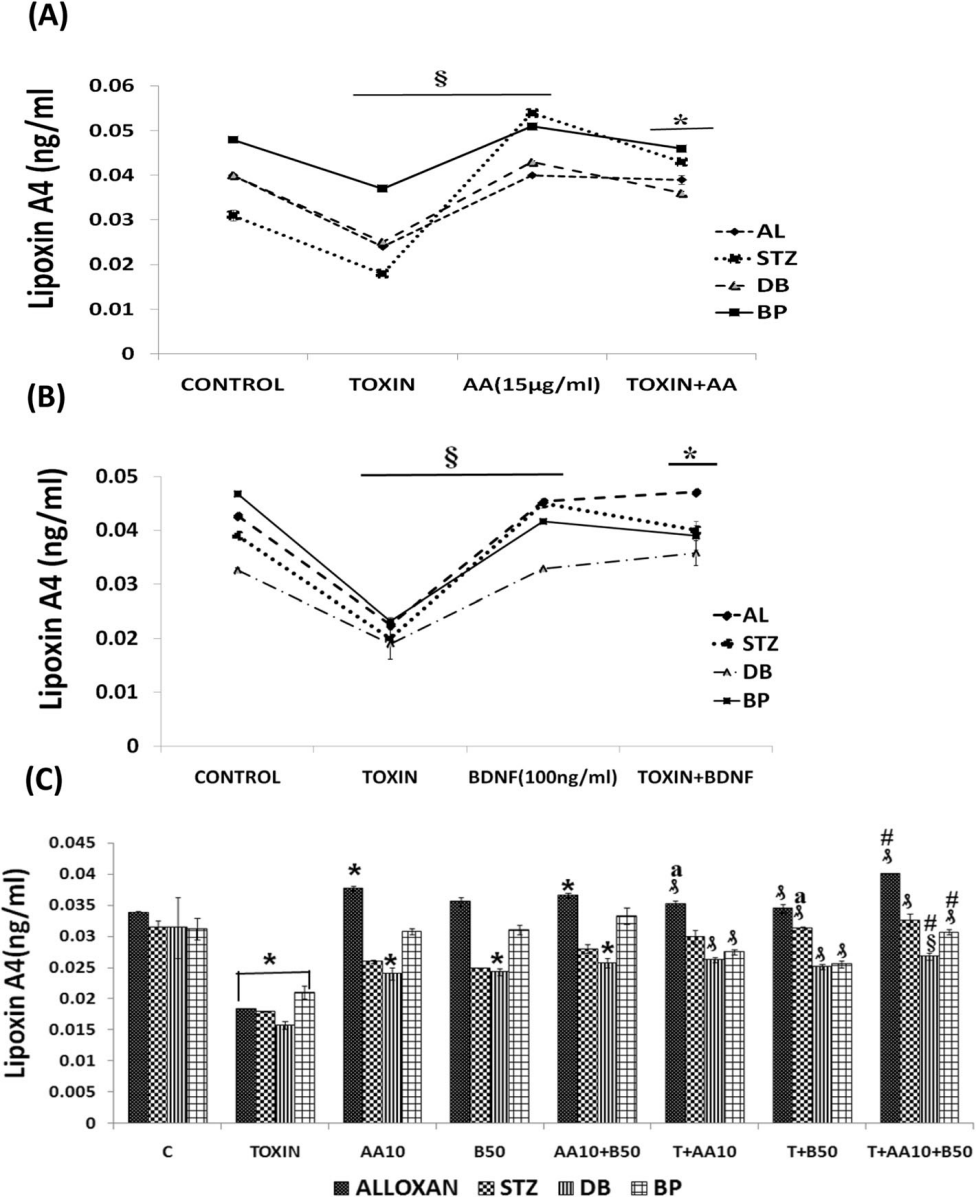
Measurement of LXA4 in the supernatants of RIN5F cells. **a**) Measurement of LipoxinA4 (ng/ml) in the supernatants of RIN5F cells treated with AL (6 mM)/ BP (1.5 mM)/STZ (20 mM)/DB (100 ng) ± AA (15 μg/ml). All values expressed as mean ± SEM. § *P* < 0.001 vs untreated control, and * *P* < 0.001 vs toxin-treated group. **b**) Measurement of LipoxinA4 (ng/ml) in the supernatants of RIN5F cells treated with AL (6 mM)/BP (1.5 mM)/STZ (20 mM)/DB (100 ng) ± BDNF (100 ng/ml). All values expressed as mean ± SEM. § *P* < 0.001 vs untreated control, and *P < 0.001 vs toxin-treated group. **c**) Measurement of LipoxinA4 (ng/ml) in the supernatants of RIN5F cells following synergistic treatment with sub-optimal dose of Arachidonic acid (AA-10 μg/ml) and BDNF (50 ng/ml) ± AL(6 mM)/BP(1.5 mM)/STZ(20 mM)/DB(100 ng/ml). All values expressed as mean ± SEM. * *P* < 0.01 vs untreated control; ₰*P* < 0.001; § *P* < 0.05 vs toxin group; ^a^*P* ≤ 0.01 vs individual and respective treated groups (i.e., AA10 vs T + AA10; B50 vs T + B50; AA10 + B50 vs T + AA10 + B50) and ^#^*P* ≤ 0.05 vs treated and synergistic combination group (i.e., T + AA10 vs T + AA10 + B50; T + B50 vs T + AA10 + B50). All the above set of experiments were done in triplicate (*n* = 3) and all values expressed as mean ± SEM. T = Toxin (AL/STZ/BP/DB)

Since various PUFAs showed cytoprotective action (Fig. 2a-d) and AL/STZ/DB/BP suppressed the production and secretion of LXA4 by RIN5F cells in vitro, we next studied whether n-6 PUFAs: AA and GLA and n-3 PUFAs: EPA/DHA can also restore LXA4 production to normal inhibited by these cytotoxins (AL/STZ/DB/BP). These studies revealed that GLA, AA, EPA and DHA (15 μg/ml) can restore to normal AL/STZ/DB/BP-induced suppression of LXA4 production by RIN5F cells (*P < 0.01 compared to untreated control and §*P* < 0.001 compared to cytotoxic chemical) as shown in Figs. 4B, 4C and 5A. Of all the PUFAs tested, AA was found to be the most potent in enhancing LXA4 secretion by RIN5F cells. These results suggest that the cytoprotective action of PUFAs can be ascribed to their ability to enhance LXA4 production by RIN5F cells.

#### BDNF restores LXA4 secretion by RIN5F cells suppressed by AL/STZ/DB/BP

Since BDNF was as effective as LXA4 in preventing the cytotoxic action of AL/BP/STZ/DB on RIN5F cells in vitro, we next tested whether BDNF modulates LXA4 secretion in these cells. It is evident from the results depicted in Fig. 5b that at the dose tested BDNF (100 ng/ml) can restore LXA4 synthesis and secretion to normal that was suppressed by AL/BP/STZ/DB.

#### Synergistic effect of sub-optimal doses of BDNF (50 ng/ml) and PUFAs (10 μg/ml) on LXA4 secretion in RIN5F

The results shown in Fig. 3a revealed that a combination of sub-optimal doses of AA and BDNF can restore viability of RIN5F cells to normal that was suppressed by AL/BP/STZ/DB. Hence, we next tested whether LXA4 secretion by RIN5F cells can be restored to normal that was suppressed by AL/BP/STZ/DB when a combination of sub-optimal doses of AA and BDNF is used. These results given in Fig. 5c showed that a combination of sub-optimal doses of AA and BDNF can indeed restore LXA4 secretion to normal by RIN5F cells in vitro.

### BDNF ELISA studies

#### Effect of AL/STZ/DB/BP on BDNF secretion by RIN5F cells

Based on the results shown in Fig. 3, it is evident that there is a close interaction among BDNF, PUFAs and LXA4. Since, BDNF can prevent apoptosis induced by AL/STZ/DB/BP, we wanted to know whether these chemicals alter the formation and secretion of BDNF by RIN5F cells in vitro*.* Pre–treatment schedule was employed to test the effect of AL/BP and simultaneous treatment schedule to test the effect of DB/STZ on BDNF secretion by RIN5F cells in vitro. Results of this study given in Table 1 revealed that AL/STZ/DB/BP significantly decreased (*P < 0.001) the formation and secretion of BDNF by RIN5F cells.

#### Effect of AA/GLA/EPA/DHA (15 μg/ml) on BDNF secretion on AL/STZ/DB/BP treated RIN5F cells

**Fig. 6:**
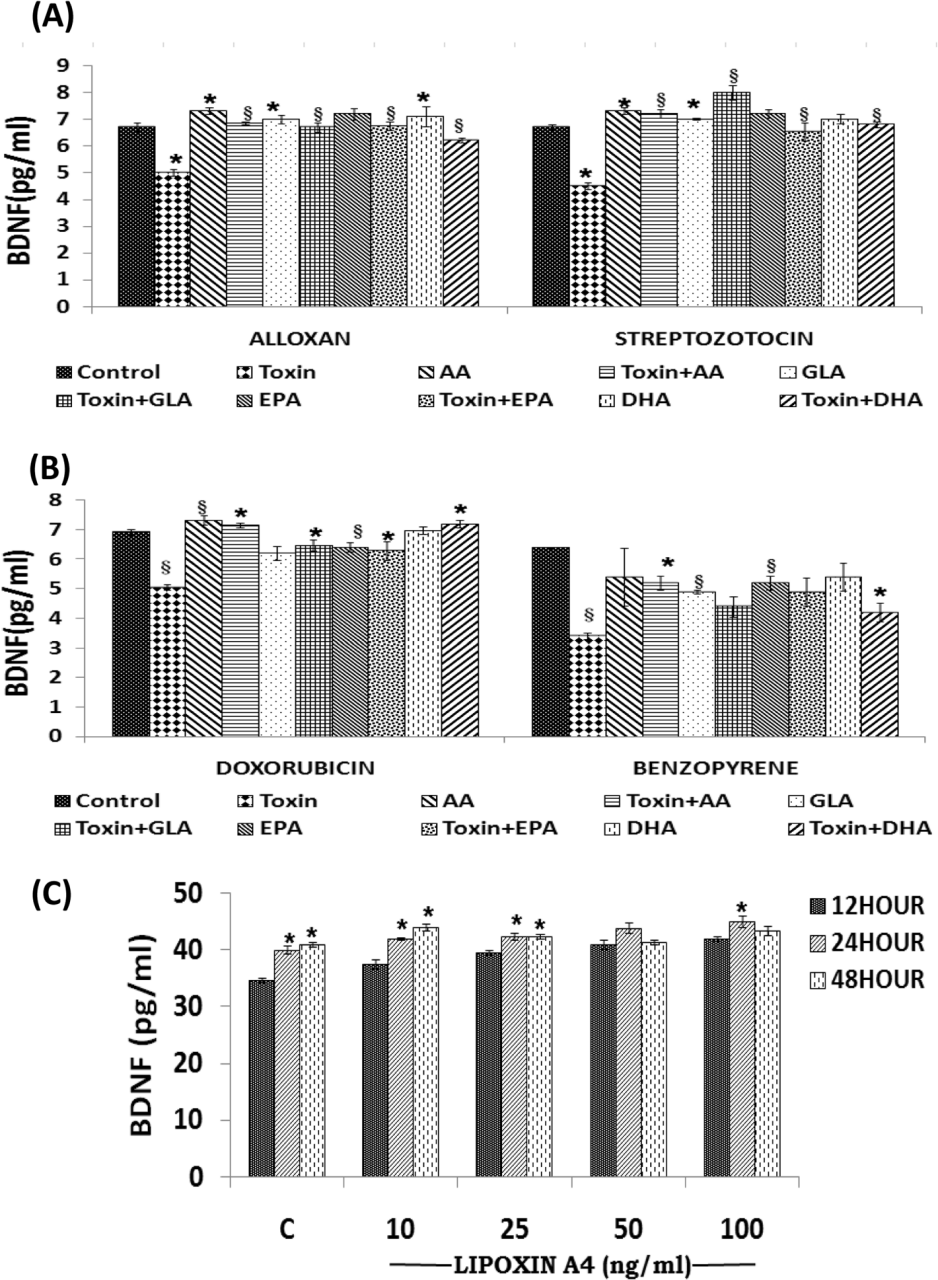
Measurement of BDNF (pg/ml) secretion by RIN5F cells in vitro. **a**) Effect of various PUFAs on the secretion of BDNF (pg/ml) by RIN5F cells in vitro that was suppressed by AL/BP. **b**) Effect of various PUFAs on the secretion of BDNF (pg/ml) by RIN5F cells in vitro that was suppressed by STZ (20 mM)/DB (100 ng/ml). All values expressed as mean ± SEM. *P < 0.01 vs untreated control § *P* < 0.001 vs toxin -treated group. AA, arachidonic acid; GLA, γ-linolenic acid; EPA, eicosapentaenoic acid; DHA, docosahexaenoic acid. **c**) Effect of LXA4 (10, 25, 50 and 100 ng/ml) at the end of 12, 24 and 48 h of supplementations on BDNF secretion by RIN5F cells in vitro. All values expressed as mean ± SEM. *P < 0.001 vs respective untreated control

We next studied whether n-6 PUFAs: AA and GLA and n-3 PUFAs: EPA/DHA can restore BDNF production to normal inhibited by cytotoxins AL/STZ/DB/BP. These studies revealed that GLA, AA, EPA and DHA (15 μg/ml) can restore to normal AL/STZ/DB/BP-induced suppression of BDNF production by RIN5F cells (*P < 0.01 as shown in Fig. 6a and b. Of all the PUFAs tested, AA was found to be the most potent in enhancing BDNF secretion by RIN5F cells. These results suggest that the cytoprotective action of PUFAs can be ascribed to their ability to enhance BDNF production by RIN5F cells.

#### Effect of LXA4 (10, 50 and 100 ng/ml) on BDNF secretion by RIN5F cells

Since both BDNF and LXA4 showed cytoprotective actions and BDNF restored LXA4 secretion to normal by AL/STZ/BP/DB-induced suppressive action, we next studied whether LXA4 is also capable of restoring BDNF secretion to normal in these cells. The results of this study given in Fig. 6c, revealed that there was a significant dose and time dependent increase ("*P < 0.001) in the levels of BDNF when compared to untreated control upon treatment with LXA4 alone. These results (see Fig. 6c) suggest that there is a close interaction between BDNF and LXA4.

**Fig. 7:**
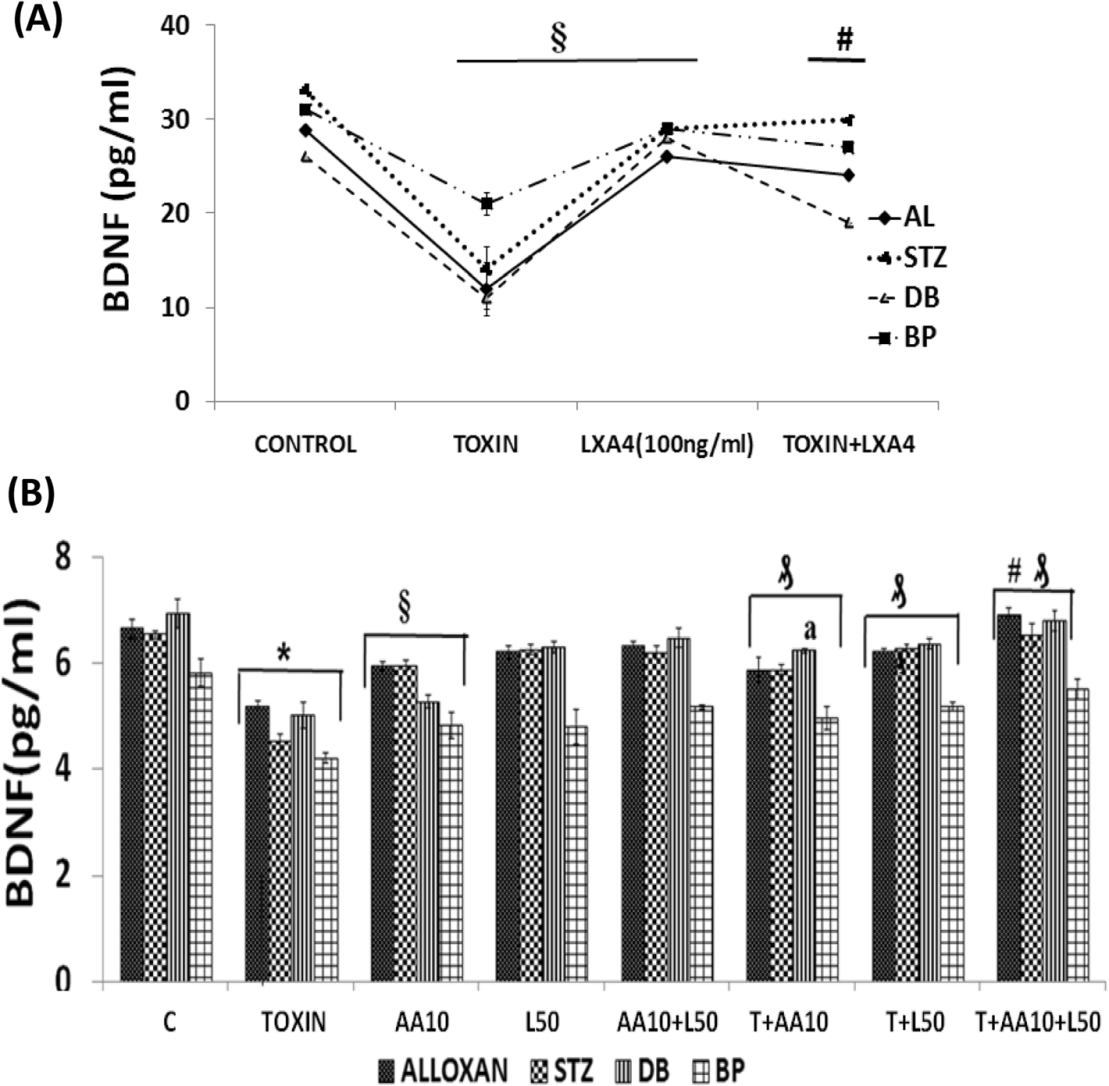
Measurement of BDNF. **a**) Effect of LXA4 (100 ng/ml) on BDNF secretion by RIN5F cells in vitro that was suppressed by AL (6 mM)/BP (1.5 mM)/STZ (20 mM)/ DB(100 ng/ml). All values expressed as mean ± SEM. § *P* < 0.001 Vs untreated control, and #*P* < 0.001 Vs toxin-treated group. **b**) Effect of combined treatment with sub-optimal doses of AA (10 μg/ml) and LXA4 (50 ng/ml) on the secretion of BDNF that was suppressed by AL (6 mM)/ BP (1.5 mM)/ STZ (20 mM)/DB (100 ng/ml) by RIN cells *invitro*. All values expressed as mean ± SEM. *P < 0.01; § *P* < 0.05 Vs untreated control; ₰*P* < 0.001 Vs toxin group; ^a^*P* ≤ 0.05: AA10 Vs T + AA10; L50 Vs T + L50; AA10 + L50 Vs T + AA10 + L50) and ^#^*P* ≤ 0.01: T + AA10 Vs T + AA10 + L50; T + B50 Vs T + AA10 + L50). All the above set of experiments were done in triplicate (*n* = 3) and all values expressed as mean ± SEM

In a further extension of this study, we also studied the effect of LXA4 on BDNF secretion by RIN5F cells in the presence of AL/STZ/BP/DB. These results (Fig. 7a) showed that LXA4 at the doses tested can restore BDNF secretion to normal that was suppressed by AL/STZ/BP/DB.

#### Effect of a combination of sub-optimal doses of AA (10 μg/ml) and LXA4 (50 ng/ml) on BDNF secretion

Next, we studied whether sub-optimal doses of AA and LXA4 can restore BDNF secretion to normal by RIN5F cells that was suppressed by AL/STZ/BP/DB. The results of this study given in Fig. 7b showed that this is indeed the case.

#### DNA fragmentation studies

**Fig. 8:**
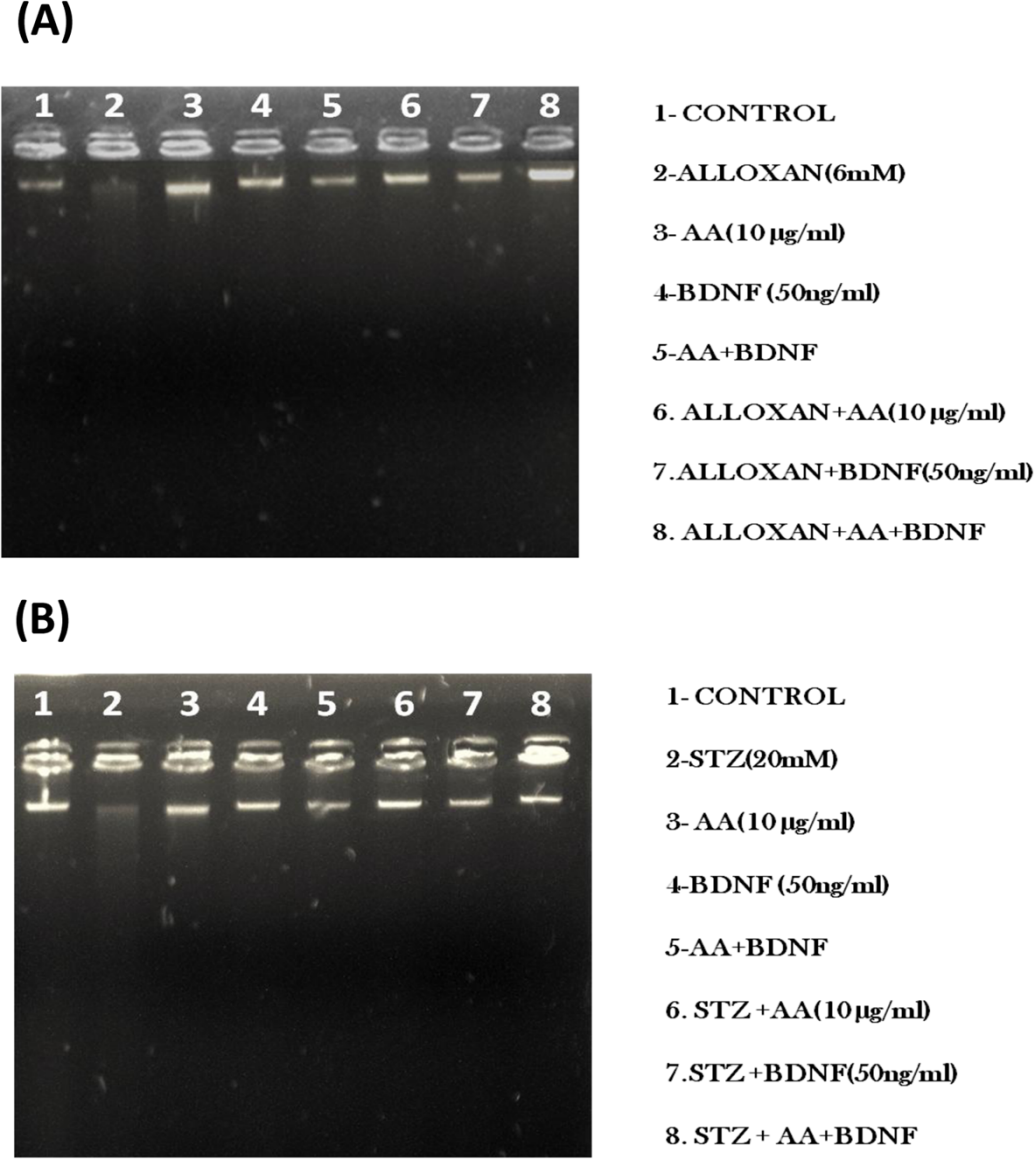
**a** Effect of BDNF (50 ng/ml) and AA(10 μg/ml) on Alloxan-induced DNA damage on RIN5F cells. Lane 1: untreated control, Lane 2: Alloxan (6 mM), Lane 3: AA(10/μg/ml), Lane 4: BDNF(50 ng/ml), Lane 5: AA+BDNF, Lane 6: AL + AA(10 μg/ml), Lane 7: AL + BDNF(50 ng/ml), Lane 8: AL + AA+BDNF(50 ng/ml). **b** Effect of BDNF (50 ng/ml) and AA(10 μg/ml) on Streptozotocin-induced DNA damage on RIN5F cells. Effect of BDNF (50 ng/ml) and AA(10 μg/ml) on Streptozotocin-induced DNA damage on RIN5F cells. Lane 1: untreated control, Lane 2: Streptozotocin (20 mM), Lane 3: AA(10/μg/ml), Lane 4: BDNF(50 ng/ml), Lane 5: AA+BDNF, Lane 6: STZ + AA(10 μg/ml), Lane 7: STZ + BDNF(50 ng/ml), Lane 8: STZ + AA(10 μg/ml), +BDNF(50 ng/ml)

Since of all the PUFAs tested AA is the most effective lipid in preventing AL/STZ/DB/BP-induced cytotoxicity to RIN5F cells, we next investigated whether AL/STZ induce apoptosis of RIN5F cells can be prevented by AA. The results of this study revealed that both AL and STZ (data with BP and DB is not shown)-induced apoptosis of RIN5F cells is prevented by AA (Fig. 8). Similar results were obtained with BP and DB and other fatty acids (GLA, EPA and DHA and LXA4 and BDNF). It was also observed that a combination of sub-optimal doses of AA (10 μg) and BDNF (50 ng) can prevent apoptosis-induced by AL and STZ of RIN5F cells (Fig. 8).

#### Antioxidant studies

To determine whether the cytoprotective action of AA and BDNF against the apoptotic actions of AL, STZ, DB and BP is due to changes in the concentrations of various anti-oxidants in RIN5F cells under the conditions studied, we estimated changes in the activities of anti-oxidants: SOD, catalase, glutathione S-transferase, glutathione peroxidase and lipid peroxides and nitric oxide in these cells. AL, STZ, DB and BP produced significant changes (* P < 0.01,# P ≤ 0.05, ** P < 0.001) in the concentrations of SOD, catalase, glutathione S-transferase, glutathione peroxidase, lipid peroxides (§ P ≤ 0.05,# P ≤ 0.02, * P < 0.01, ** *P < 0.001)* and nitric oxide in RIN5F cells as shown in Tables 2, 3, 4, 5. Almost all these changes induced by AL, STZ, DB and BP reverted to normal or close to normal when treated with BDNF (** P < 0.001) and AA alone or in combination (AA + BDNF).

**Table 2:** Summary of analysis of various anti-oxidants in RIN5F treated with Alloxan(Pre-treatment)/STZ (Sim-treatment) and sub-optimal doses of AA (10 μg/ml) and BDNF (50 ng/ml)

SUMMARY OF THE ANALYSIS OF VARIOUS ANTI-OXIDANT ENZYMES IN RIN5F CELLS TREATED WITH AL/STZ					
CHEMICAL	GROUP	SOD (Units/mg protein)	CAT (Mm H2O2/min/mg protein	GST (mM/min/gm protein)	GPX (mM/min/gm protein)
ALLOXAN	CONTROL	23.6 ± 1.7	466 ± 52.1	54.75 ± 1.4	739.8 ± 37.2
	AL(6 mM)	39.88 ± 3.5^§^	739.1 ± 45^§^	56.85 ± 3.2^§^	1649 ± 81.6^§^
	AA(10 μg/ml)	19.21 ± 0.9	407.7 ± 36.5	47.74 ± 1.8	534.79 ± 24.4
	BDNF(50 ng/ml)	19.12 ± 1.4	384.4 ± 30.9	41.44 ± 2.2	556 ± 18.7
	AA + BDNF	21.58 ± 2.6	326.7 ± 24.9	38.79 ± 2.1	560 ± 16.6
	AL + AA(10 μg/ml)	21.28 ± 2.8	356 ± 67	44 ± 0.86*	495.3 ± 7.4
	AL + BDNF	26 ± 1.43^#^	459 ± 76.2^#^	45.5 ± 0.8^#^	503.6 ± 3.5^#^
	AL + AA + BDNF	29.25 ± 0.7	432.3 ± 60	54.4 ± 2.23	768 ± 23.6
STREPTOZOTOCIN	CONTROL	24 ± 1.6	503.9 ± 45.2	54.5 ± 0.1	701 ± 19.4
	STZ(20 mM)	39.76 ± 1.4^§^	791 ± 86.1^§^	78.5 ± 0.76 ^§^	1036 ± 38.3^§^
	AA(10 μg/ml)	21 ± 1.2	446 ± 49.3	51.9 ± 0.2	584 ± 23.1
	BDNF(50 ng/ml)	20.6 ± 1.3	420 ± 41.8	45.5 ± 0.42	581 ± 30.1
	AA + BDNF	24.1 ± 3.1	366 ± 32.2	43.7 ± 0.16	550 ± 38.6
	STZ + AA(10 μg/ml)	25 ± 7.8	528 ± 67	48.2 ± 0.96	505.3 ± 7.4
	STZ + BDNF	32 ± 1.7^#^	559 ± 86.2^#^	50.5 ± 0.2^#^	603.6 ± 3.5^#^
	STZ + AA + BDNF	34 ± 1.08	656 ± 57.1	54.5 ± 0.08	752.1 ± 21.4

Superoxide dismutase (SOD) is expressed as U SOD/mg of protein; Catalase (CAT) is expressed as μM of H_2_O_2_ consumed/minute/mg of protein; Glutathione-S-transferase (GST) is expressed as μM conjugate formed/ minute/gm of protein; Glutathione peroxidase (GPX) is expressed as μg of glutathione consumed/minute/gm of protein. *₰P < 0.001*, *§ P ≤ 0.01* Vs Untreated control and * P < 0.01,# P ≤ 0.05, ** P < 0.001 Vs AL/STZ -treated group

**Table 3:** Summary of Lipid peroxides and nitric oxide analysis in RIN5F cells treated with Alloxan (Pre-treatment) /STZ(Simultaneous) and suboptimal doses of AA (10 μg/ml) + BDNF (50 ng/ml)

SUMMARY OF THE ANALYSIS OF LIPID PEROXIDES AND NITRIC OXIDE LEVELS IN RIN5F CELLS TREATED WITH AL/STZ					
CHEMICAL	GROUP	LIPID PEROXIDES (μM TMOP)		NITRIC OXIDE (μM Nitrite)	
		Supernatant Lysates		Supernatant Lysates	
ALLOXAN	CONTROL	0.47 ± 0.04	1.2 ± 0.04	0.58 ± 0.03	1.2 ± 0.03
	AL(6 mM)	0.8 ± 0.04*	1.6 ± 0.09*	0.87 ± 0.05	1.4 ± 0.05*
	AA(10 μg/ml)	0.45 ± 0.06	1.1 ± 0.02	0.54 ± 0.06	1.15 ± 0.03
	BDNF(50 ng/ml)	0.44 ± 0.03	1.3 ± 0.05	0.43 ± 0.03	1.18 ± 0.07
	AA + BDNF	0.37 ± 0.03	1.3 ± 0.1	0.49 ± 0.04	1.16 ± 0.05
	AL + AA(10 μg/ml)	1.2 ± 0.03^#^	1.3 ± 0.05^#^	1.42 ± 0.03 ^#^	1.16 ± 0.05 ^#^
	AL + BDNF	0.9 ± 0.03^§^	0.9 ± 0.05^§^	0.94 ± 0.03^§^	1.25 ± 0.02^§^
	AL + AA + BDNF	0.74 ± 0.06	1.4 ± 0.05	0.89 ± 0.07	1.12 ± 0.05
STREPTOZOTOCIN	CONTROL	0.68 ± 0.05	1.3 ± 0.04	0.64 ± 0.04	1.08 ± 0.05
	STZ(20 mM)	0.89 ± 0.03	1.6 ± 0.09	0.83 ± 0.04	1.52 ± 0.06
	AA(10 μg/ml)	0.55 ± 0.03	1.2 ± 0.02	0.61 ± 0.05	1.11 ± 0.02
	BDNF(50 ng/ml)	0.54 ± 0.01	1.2 ± 0.03	0.57 ± 0.02	1.09 ± 0.02
	AA + BDNF	0.51 ± 0.03	1.2 ± 0.03	0.58 ± 0.01	1.11 ± 0.05
	STZ + AA(10 μg/ml)	0.72 ± 0.06^#^	0.9 ± 0.09^#^	0.79 ± 0.07^#^	1.09 ± 0.04^#^
	STZ + BDNF	0.66 ± 0.08^§^	0.8 ± 0.09^§^	0.69 ± 0.4^§^	0.9 ± 0.08^§^
	STZ + AA + BDNF	0.59 ± 0.03	1.3 ± 0.03	0.65 ± 0.0	1.15 ± 0.05

Lipid peroxides formed are expressed as μmoles of TMOP formed; Nitric oxide formed is expressed as μ moles of nitrite formed. *P < 0.001 Vs Untreated control and § P ≤ 0.05, # P ≤ 0.02, * P < 0.01, ** *P < 0.001* Vs AL/STZ -treated group

**Table 4:** Summary of analysis of various anti-oxidants in RIN5F treated with Benzopyrene(Pre-treatment)/DB(Sim-treatment) and sub-optimal doses of AA(10 μg/ml) + BDNF(50 ng/ml)

SUMMARY OF THE ANALYSIS OF VARIOUS ANTI-OXIDANT ENZYMES IN RIN5F CELLS TREATED WITH DB/BP					
CHEMICAL	GROUP	SOD (Units/mg protein)	CAT (Mm H2O2/min/mg protein	GST (mM/min/gm protein)	GPX (mM/min/gm protein)
DOXORUBICIN	CONTROL	34.6 ± 0.8	369 ± 58.6	44.75 ± 3.4	686.8 ± 27.2
	DB(100 ng/ml)	29.2 ± 1.5^§^	689.1 ± 28^§^	51.65 ± 2.2^§^	1845 ± 69^§^
	AA(10 μg/ml)	31.2 ± 0.4	414.6 ± 26.5	37.74 ± 1.8	519 ± 14.8
	BDNF(50 ng/ml)	21 ± 0.9^₰^	404 ± 29.9	38.84 ± 2.9	601 ± 32.4
	AA + BDNF	29.5 ± 2.9	386 ± 32	32.49 ± 2.1	515 ± 21.4
	DB + AA(10 μg/ml)	30.6 ± 1.8	323 ± 41	39.3 ± 1.2	504 ± 5.4
	DB + BDNF	21 ± 2.8^#^	409 ± 58.2	41.2 ± 0.8^#^	698 ± 5.9^#^
	DB + AA + BDNF	32.25 ± 0.3	414.3 ± 58	44.4 ± 2.28	727 ± 33
BENZOPYRENE	CONTROL	44 ± 1.6	515.9 ± 49	44.5 ± 0.1	791 ± 29
	BP(1.5 mM)	31.2 ± 1.8^§^	801 ± 61^§^	82.34 ± 1.2 ^§^	997 ± 41.3^§^
	AA(10 μg/ml)	19 ± 1.2	487 ± 57	48.9 ± 0.8	504 ± 33.3
	BDNF(50 ng/ml)	18.6 ± 1.7	402 ± 24.9	49 ± 0.62	506 ± 39.3
	AA + BDNF	22 ± 3.1	387 ± 38.2	41.7 ± 0.1	567 ± 39.1
	BP + AA(10 μg/ml)	24 ± 8.2	514 ± 39.8	51.2 ± 1.2	798.3 ± 2.6
	BP + BDNF	31 ± 2.1^#^	498 ± 74.8^#^	48.5 ± 0.2^#^	799.6 ± 3.5^#^
	BP + AA + BDNF	39 ± 0.67	508 ± 57.1	52.5 ± 0.08	698.1 ± 17.8

Superoxide dismutase (SOD) is expressed as U SOD/mg of protein; Catalase (CAT) is expressed as μM of H_2_O_2_ consumed/minute/mg of protein; Glutathione-S-transferase (GST) is expressed as μM conjugate formed/ minute/gm of protein; Glutathione peroxidase (GPX) is expressed as μg of glutathione consumed/minute/gm of protein. *₰ P < 0.05*, *§ P ≤ 0.01* Vs Untreated control and * P < 0.01, # P ≤ 0.05, ** P < 0.001 Vs DB/BP -treated group

**Table 5:** Summary of Lipid peroxides and nitric oxide analysis in RIN5F treated with BP(Pre-treatment) /DB(Simultaneous) and suboptimal doses of AA (10 μg/ml) + BDNF(50 ng/ml)

SUMMARY OF THE ANALYSIS OF LIPID PEROXIDES AND NITRIC OXIDE LEVELS IN RIN5F CELLS TREATED WITH DB/BP					
CHEMICAL	GROUP	LIPID PEROXIDES (μM TMOP)		NITRIC OXIDE (μM Nitrite)	
		Supernatant Lysates		Supernatant Lysates	
DOXORUBICIN	CONTROL	0.43 ± 0.03	1.1 ± 0.03	0.71 ± 0.02	0.9 ± 0.03
	DB(100 ng/ml)	0.75 ± 0.02	1.8 ± 0.04	0.91 ± 0.04	1.2 ± 0.03
	AA(10 μg/ml)	0.23 ± 0.02	1.1 ± 0.03	0.49 ± 0.02^§^	1.2 ± 0.02^§^
	BDNF(50 ng/ml)	0.37 ± 0.02	1.3 ± 0.04	0.41 ± 0.01	1.2 ± 0.06
	AA + BDNF	0.48 ± 0.01	1.1 ± 0.09	0.39 ± 0.01	1.2 ± 0.04
	DB + AA(10 μg/ml)	0.55 ± 0.04^#^	1.2 ± 0.05	1.02 ± 0.02^#^	1.1 ± 0.04
	DB + BDNF	0.61 ± 0.03^§^	0.87 ± 0.02^§^	0.91 ± 0.01^§^	0.88 ± 0.08^§^
	DB + AA + BDNF	0.59 ± 0.03	1.2 ± 0.06	0.79 ± 0.03	1.1 ± 0.05
BENZOPYRENE	CONTROL	0.58 ± 0.02	0.8 ± 0.02	0.74 ± 0.02	0.98 ± 0.05
	BP(1.5 mM)	0.99 ± 0.01	1.6 ± 0.03	0.99 ± 0.03	1.4 ± 0.06
	AA(10 μg/ml)	0.61 ± 0.04	1.1 ± 0.03	0.69 ± 0.01	1.11 ± 0.02
	BDNF(50 ng/ml)	0.59 ± 0.02	0.89 ± 0.01	0.61 ± 0.03	1.07 ± 0.03
	AA + BDNF	0.61 ± 0.01	1.2 ± 0.02	0.72 ± 0.01	1.09 ± 0.04
	BP + AA(10 μg/ml)	0.69 ± 0.05^#^	0.89 ± 0.04^#^	0.69 ± 0.07^#^	1.05 ± 0.02^#^
	BP + BDNF	0.72 ± 0.03^§^	0.7 ± 0.05^§^	0.71 ± 0.4^§^	1.1 ± 0.07^§^
	BP + AA + BDNF	0.64 ± 0.06	1.1 ± 0.02	0.79 ± 0.01	1.23 ± 0.03

Lipid peroxides formed are expressed as μmoles of TMOP formed; Nitric oxide formed is expressed as μ moles of nitrite formed. *P < 0.01 Vs Untreated control and § P ≤ 0.05, # P ≤ 0.02, * P < 0.01, ** *P < 0.001* Vs DB/BP -treated group

#### Plasma concentrations of BDNF and LXA4 in STZ-induced type 2 DM animals

**Fig. 9:**
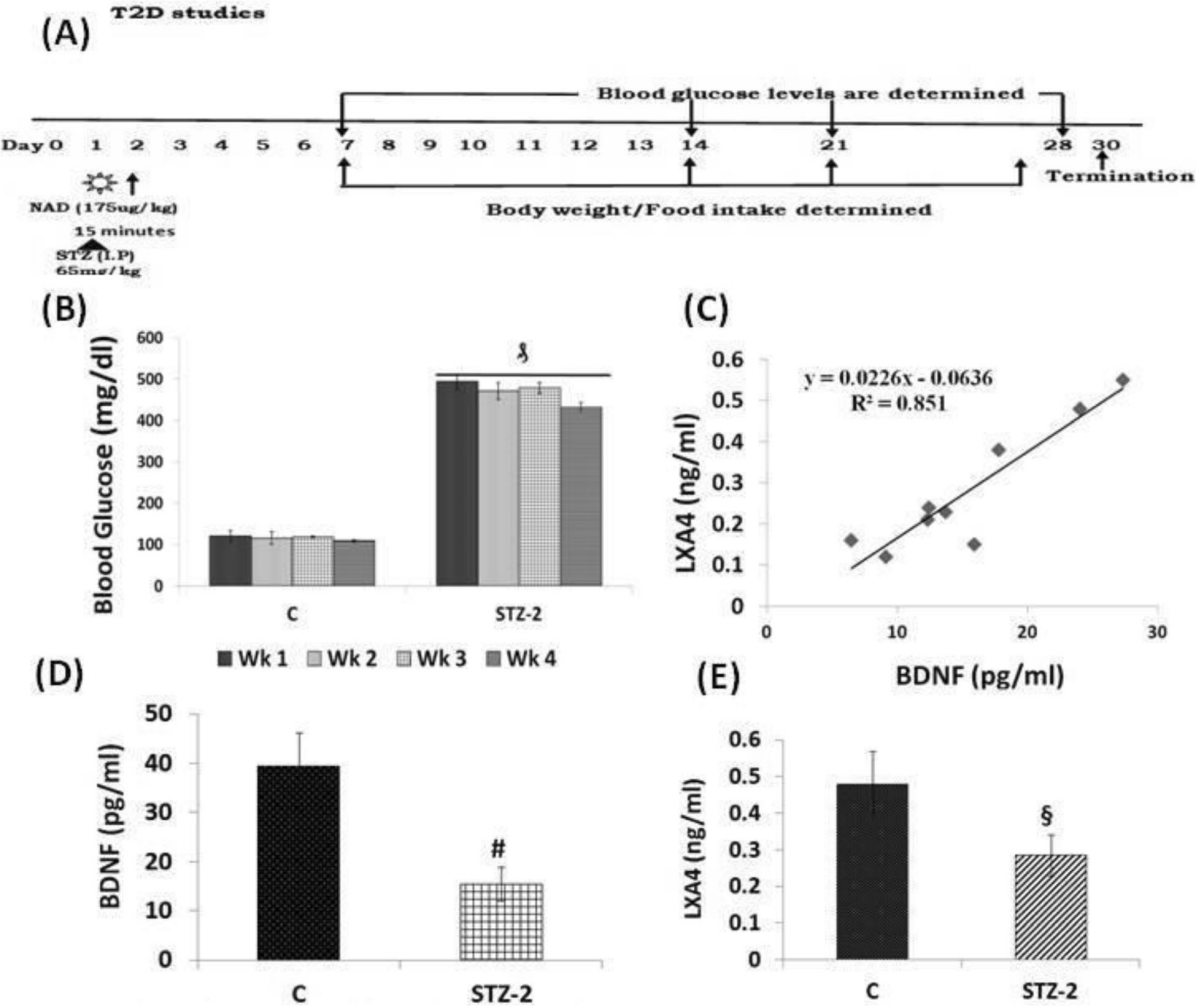
**a** STZ-induced type 2 diabetes protocol. Animals were housed for 1 week for acclimatization after which single I.P injection of STZ was given and blood glucose levels were estimated once in 10 days till the end of the study. **b** Blood glucose levels were measured once in 7 days until the end of the study. ₰*P* < 0.001 vs control group. **c** Correlation analysis of levels of BDNF and LXA4 in the plasma of STZ treated and control animals (*n* = 8) regression coefficient R = 0.851. **d** Plasma LXA4 levels (ng/ml) in STZ-induced type 2 diabetes mellitus Wistar rats on day 30 of the study. All values expressed as mean **±** SEM. § *P* < 0.001 vs untreated control group of animals. **e** Plasma BDNF levels (pg/ml) in STZ-induced type 2 diabetes mellitus Wistar rats on day 30 of the study. All values expressed as mean **±** SEM. # *P* < 0.001 vs untreated control

To verify whether the above described in vitro results are relevant to an in vivo situation, we measured the plasma levels of LXA4 and BDNF in STZ-induced type 2 DM in Wistar rats. The protocol of this study is shown in Fig. 9a. Fasting blood glucose levels were significantly (*P* < 0.05) increased from day 2 following the administration of STZ (Fig. 9b) that persisted when measured on 10th, 20th and 30th days of the study confirming the development of type 2 DM in these animals. Simultaneously, body weight was reduced, and food consumption was increased in these STZ-induced type 2 DM Wistar rats (data not shown).

Plasma BDNF and LXA4 levels were significantly lower in STZ-induced type 2 DM animals compared to control (Fig. 9c-d). A direct correlation between plasma LXA4 and BDNF levels in these type 2 DM animals was noticed (Fig. 9b). These results are in tune with the in vitro results wherein it was observed that LXA4 and BDNF levels in the supernatant of AL/STZ/BP/DB-treated RIN5F cells were low (see Fig. 4a-d and Fig. 6a-d). These results suggest that AL/STZ/BP/DB inhibit the production and secretion of LXA4 and BDNF by pancreatic beta cells both in vitro and in vivo and thus, produce their cytotoxic actions.

## Discussion

The results of the present study showed that AL/STZ/DB/BP-induced cytotoxicity/apoptosis to RIN5F cells in vitro can be prevented by BDNF and various PUFAs, especially AA, and LXA4, an anti-inflammatory metabolite of AA (Figs. 1 and 2). It is noteworthy that a combination of sub-optimal doses of BDNF and AA/EPA/LXA4 protected RIN5F cells against the cytotoxic actions of AL/STZ/DB/BP (Fig. 3), suggesting that a combination of PUFAs and LXA4, BDNF and PUFAs, and LXA4 and BDNF is better than when these molecules are used individually. All the four chemicals tested (AL/STZ/DB/BP) suppressed the secretion of BDNF and LXA4 by RIN5F cells, suggesting that one mechanism by which these chemicals induce apoptosis is by interfering with the formation and secretion of these cytoprotective molecules (BDNF and LXA4). A combination of suboptimal doses of AA and BDNF restored the altered antioxidant status to normal (Tables 2, 3, 4, 5) indicating that PUFAs and BDNF prevent AL/STZ/DB/BP-induced apoptosis of RIN5F cells in vitro, at least, in part, by restoring oxidative stress to normal. In addition, the present study showed that STZ-induced type 2 DM is associated with low plasma BDNF and LXA4 levels (see Fig. 9). This lends support to the concept that a deficiency of BDNF and LXA4 play a significant role in the development of diabetes mellitus.

The results of the present study are in tune with our previous finding that BDNF, PUFAs and LXA4 are capable of restoring oxidant-antioxidant homeostasis to normal in RIN5F cells that is altered by AL/STZ/DB/BP treatment [25–28]. We observed that expression of BCL2, BAX and NF-κB, IKB-β and Pdx-1 that were tilted more towards pro-apoptosis and pro-inflammatory status can be restored to normal by treatment with BDNF, AA and LXA4 [25–28]. Though in the present study, we did not measure these indices (BCL2, BAX and NF-κB, IKB-β and Pdx-1) [29, 30], it is likely that restoration of BCL2, BAX and NF-κB, IKB-β and Pdx-1 expressions to normal could be responsible for the beneficial action of BDNF, AA and other PUFAs and LXA4 noted in the present study.

Both AL and STZ accumulate in pancreatic β cells by being transported by GLUT-2 receptors and bring about their cytotoxic actions on these cells by a free radical dependent process [31–33] and consequent massive increase in cytosolic calcium concentrations. The results of the present study revealed that AL brings about its action predominantly by altering superoxide anion (since an increase in SOD was seen that could be because of its feedback upregulation due to an increase in the generation of superoxide anion by AL), whereas STZ enhanced the production of both superoxide anion and hydrogen peroxide radicals (Table 2), which are in support of the known actions of AL and STZ [27–31]. Furthermore, both AL and STZ enhanced the formation of lipid peroxides and nitric oxide (see Table 2) both in the cell lysates and their supernatants suggesting that these two chemicals have significant pro-oxidant actions. On the other hand, both DB and BP decreased SOD and but enhanced those of GST, catalase and GPX in RIN5F cells and at the same time enhanced the formation of lipid peroxides and nitric oxide both in the cell lysates and their supernatants (see Tables 4 and 5), indicating that DB and BP enhance free radical generation and antioxidant content (as a consequence of feedback upregulation due to an increase in the generation of free radicals) by treated RIN5F cells that is somewhat similar to the actions of AL and STZ. These changes in the antioxidants and lipid peroxides and nitric oxide reverted to near normal in the presence of a combination of sub-optimal doses of AA and BDNF (see Tables 2, 3, 4, 5). In a previous study, we noted that AL/STZ/DB/BP-induced changes in antioxidants and lipid peroxides and nitric oxide are restored to normal by optimal doses of BDNF, AA and its metabolite LXA4 [23, 27–29] suggesting that AA and BDNF (when each chemical is used individually in optimal doses or when their suboptimal doses are given in combination) are capable of restoring oxidant-antioxidant balance to normal. These results imply that sub-optimal doses of AA and BDNF can produce the same amount of beneficial action of optimal doses of either BDNF or AA to protect RIN5F cells.

In a further extension of our previous studies [25, 29–31], in the present investigation, we evaluated the effect of AL/STZ/B/BP on the secretion of LXA4, a potent anti-inflammatory metabolite of AA. These studies showed that all the four chemicals tested are potent inhibitors of LXA4 secretion by RIN5F cells (see Figs. 4, 5, 6 and 7). It is noteworthy that AA and other PUFAs can restore to normal LXA4 secretion that was suppressed by AL/STZ/DB/BP (Fig. 4). In addition, BDNF showed unique ability to restore LXA4 secretion to normal by RIN5F cells that was suppressed by AL/STZ/DB/BP. In a similar fashion, BDNF secretion that was suppressed by AL/STZ/DB/BP was restored to normal by AA and other PUFAs and by LXA4 (Figs. 6, 7, and 8). In addition, sub-optimal doses of AA and BDNF and sub-optimal doses of AA and LXA4, when added together, restored to normal the secretion of LXA4 and BDNF respectively by RIN5F cells that was suppressed by AL/STZ/DB/BP (See Figs. 5 and 7). In this context, it is relevant to note that both BDNF and LXA4 enhanced the secretion of LXA4 and BDNF respectively by RIN5F cells in vitro (Figs. 4a and 6c) suggesting that there is a close interaction between BDNF and LXA4 and that they enhance each other’s secretion and action. In addition, other PUFAs tested (such as GLA, EPA and DHA though these fatty acids were less potent compared to AA in enhancing LXA and BDNF formation and secretion) increased the formation and secretion of both LXA4 and BDNF. These results suggest that PUFAs, LXA4 and BDNF interact with each other rather closely and restore each other’s concentrations to normal under adverse conditions (such as exposure to AL/STZ and drugs such as doxorubicin and environmental pollutants such as BP) to protect cells from their cytotoxic actions. These results are further supported by our previous bioinformatics study wherein we noted that various PUFAs and LXA4 interact rather closely with BDNF [34]. The ability of GLA, EPA and DHA to enhance LXA4 secretion by RIN5F cells (Fig. 4b and c). is rather surprising and intriguing. It is possible that GLA, EPA and DHA can displace AA from the cell membrane lipid pool and thus, enhance the formation of LXA4 from the released AA.

The observation that STZ-induced type 2 DM is associated with low plasma levels of BDNF and LXA4 (see Fig. 9) further lends support to the concept that a deficiency of these endogenous molecules could predispose to the development of diabetes mellitus. These results are interesting since, exercise that has several beneficial actions is known to enhance the formation of BDNF and LXA4 [35, 36]. It is likely that this may account for its (exercise) beneficial actions seen in improving memory, reduce risk of cardiovascular diseases and other diseases. The argument that an increase in LXA4 levels may underlie the beneficial action of exercise is likely since LXA4 is a potent vasodilator, has significant anti-inflammatory actions and suppresses the production of free radicals and pro-inflammatory cytokines IL-6 and TNF-α both in vitro and in vivo [37, 38]. Our previous studied showed that LXA4 can prevent the development of chemical-induced type 1 and type 2 DM in experimental animals [29, 30, 39]. On the other hand, BDNF is a neurotrophic molecule that is known to enhance memory especially after exercise [40, 41]. In addition, BDNF is known to be beneficial in obesity and has anti-diabetic actions [42, 43]. Since memory formation needs generation of new neurons and synapse formation that, in turn, calls for increased blood supply, it stands to reason to suggest that BDNF need to enhance LXA4 formation locally to promote angiogenesis, Thus, both BDNF and LXA4 may enhance each other’s synthesis, secretion and action that may explain the interaction between BDNF and LXA4 observed in the present study. Furthermore, the observation that BDNF and LXA4/PUFAs have synergistic actions in preventing the cytotoxic actions of AL/STZ/DB/BP on RIN5F cells (see Fig. 3) implies that even under circumstances wherein there is a partial deficiency of either BDNF or LXA4/PUFAs, the other molecule can ensure cell viability and function.

Results of our studies presented here and elsewhere [29, 30, 39] suggest that LXA4 and AA have anti-diabetic actions like BDNF that also is anti-diabetic in nature highlighting the possibility that BDNF and LXA4 support each other’s action to bring about their anti-diabetic actions as well. This argument is further supported by the fact that exercise that is beneficial in preventing obesity and development of type 2 diabetes enhances the synthesis and secretion of both BDNF and LXA4 [35, 36]. Based on these evidences, it is reasonable to propose that BDNF and LXA4 and other PUFAs function as one unit that are interdependent on each other and at the same time supportive of each other’s action. This is thus, a classic example of protein-lipid interaction that is designed for the benefit of the body. In the light of this, it can be argued that there could exist several such situations wherein there is a close interaction between proteins and lipids that calls for additional studies, which are underway in our laboratory.

Despite the interesting results, we would like to address one limitation of the present study. Though we did observe that AL/STZ/DB/BP are able to reduce the production of BDNF and LXA4 by RIN5F cells, we have not studied the exact mechanism(s) by which they are able to bring about this action. For instance, it is yet to be established whether AL/STZ/DB/BP can act on COX-2 and 5-, 12- and 15-LOX enzymes that are involved in the synthesis of LXA4. Similarly, it is not known whether AL/STZ/DB/BP suppress the production of BDNF by RIN5F cells by acting at the gene level. These studies are now underway in our lab.

## Conclusion

Based on the results of the present and previous studies it is evident that BDNF, EPA, DHA, AA, GLA and LXA4 prevent cytotoxic action of AL, STZ, DB and BP against rat pancreatic β cells and possibly, other normal cells. This cytoprotective action of BDNF, EPA, DHA, AA, and GLA could be attributed to their ability to augment the production of cytoprotective and anti-inflammatory molecule LXA4 by RIN5F cells. AL, STZ, DB and BP seem to bring about their cytotoxic action by suppressing the production of LXA4 by RIN5F cells. A combination of minimal doses of various PUFAs (10 μg/ml) and LXA4 and BDNF (50 ng/ml) showed synergistic action in protecting RIN5F cells against the cytotoxic action of AL, STZ, DB and BP. Plasma levels of BDNF and LXA4 are low in Wistar rats that were induced to develop type 2 diabetes mellitus by STZ. In summary these results suggest that there is a close interaction between various PUFAs (including LXA4) and BDNF and these molecules may function as endogenous cytoprotective and anti-diabetic molecules.

## Supplementary information

**Additional file 1.** **Figure S1:** Effect of various doses of AL, STZ, DB and BP on the viability of RIN5F cells in vitro.

**Additional file 2.** **Figure S2:** Effect of various dsoes of BDNF on the viabiltiy of RIN5F cells in vitro. 

**Additional file 3.** **Figure S3:** Effect of various doses of PUFAs and LXA4 on the viability of RIN5F cells in vitro.

## Data Availability

All the data of the present work is given in the manuscript. Any additional data and material are available on request.

## Abbreviations

AA: Arachidonic acid
AHR: Arylhydrocarbon receptor
AL: Alloxan
ALA: Alpha-linolenic acid
ARNT: AHR nuclear translocator
BDNF: Brain derived neurotrophic factor
BP: Benzo(a)pyrene
BPA: Bisphenol A
CAT: Catalase
CYP1A1: Intestinal cytochrome P450 subclass 1A1
DB: Doxorubicin
DGLA: Dihomo-gamma-linolenic acid
DHA: Docosahexaenoic acid
ELISA: Enzyme linked immunosorbent assay
EPA: Eicosapentaenoic acid
GLA: Gamma-linolenic acid
GPX: Glutathione peroxidase
GST: Glutathione-S-transferase
LA: Linoleic acid
LXA4: Lipoxin A4
MTT: 3-(4,5-dimethylthiazole-2-yl)-2,5-diphenyltetrazolium bromide
PAHs: Polycyclic aromatic hydrocarbons
PCBs: Polychlorinated biphenyls
PPAR: Peroxisome proliferator-activated receptor
PUFAs: Polyunsaturated fatty acids
RIN5F cells: Rat insulinoma cells
ROS: Reactive oxygen species
SOD: Superoxide dismutase
STZ: Streptozotocin
TLR2: Toll-like receptor 2
Type 2 DM: Type 2 diabetes mellitus
