# Supplementary figures and images for: PUFAs, BDNF and lipoxin A4 inhibit chemical-induced cytotoxicity of RIN5F cells in vitro and streptozotocin-induced type 2 diabetes mellitus in vivo
Source: Lipids Health Dis. 2019 Dec 10;18:214. doi: 10.1186/s12944-019-1164-7 (PMC7159172; doi:10.1186/s12944-019-1164-7)

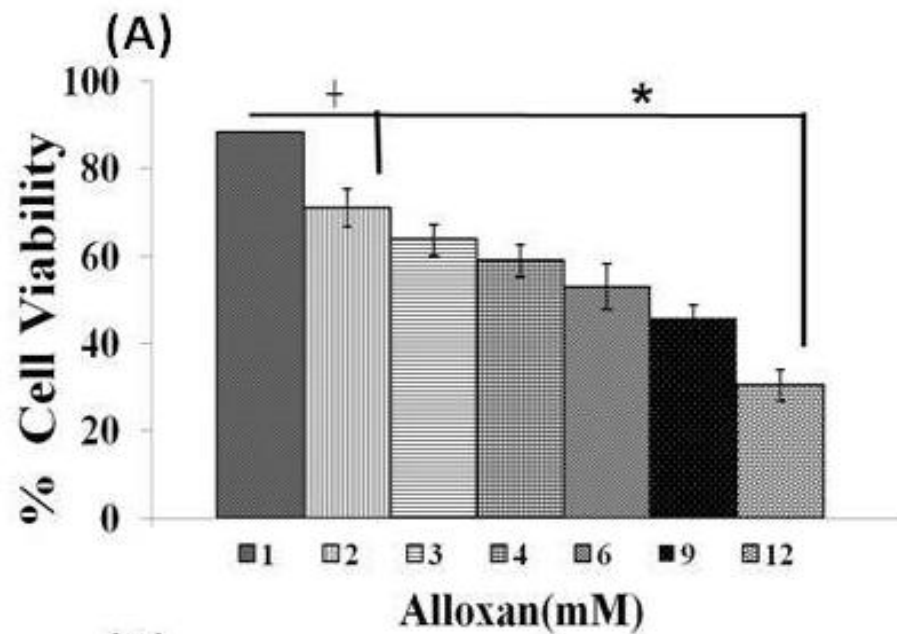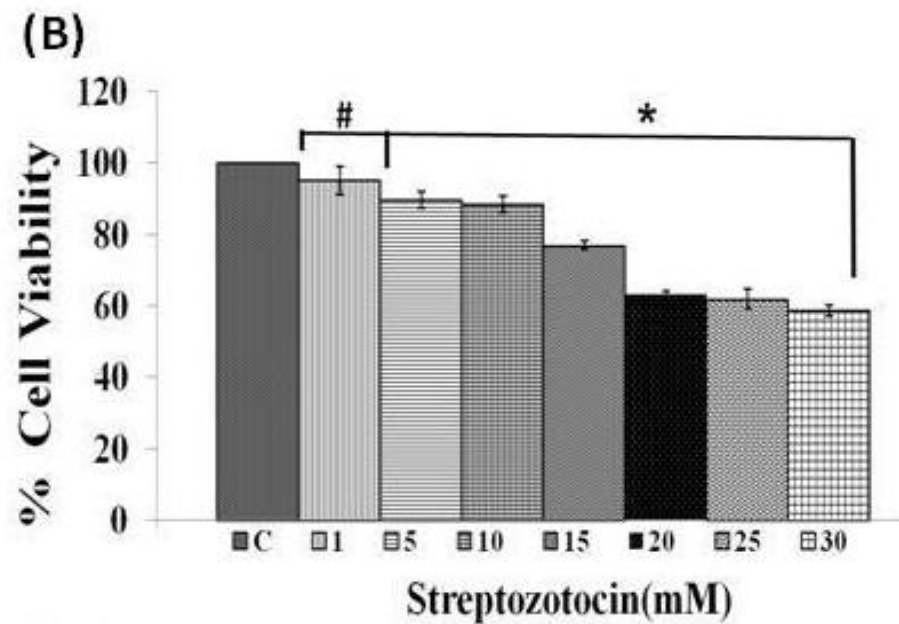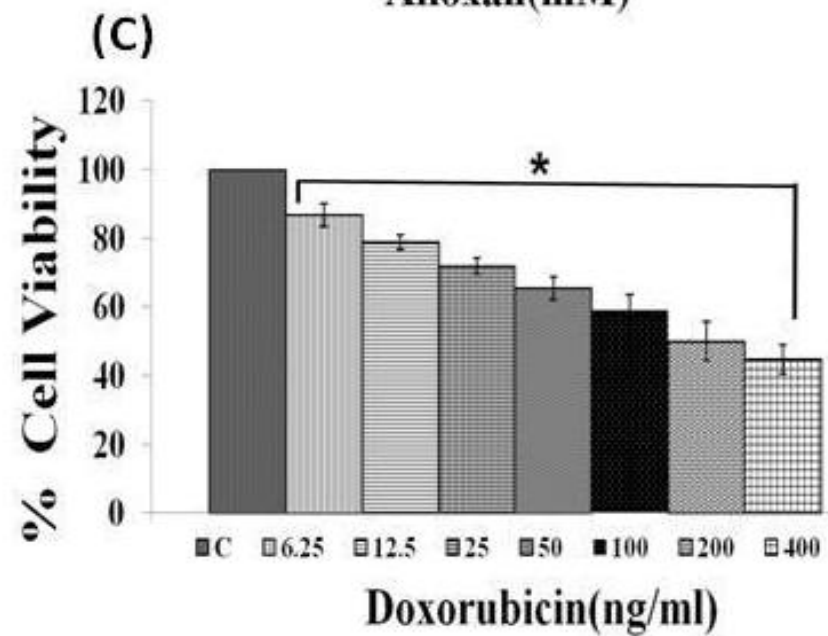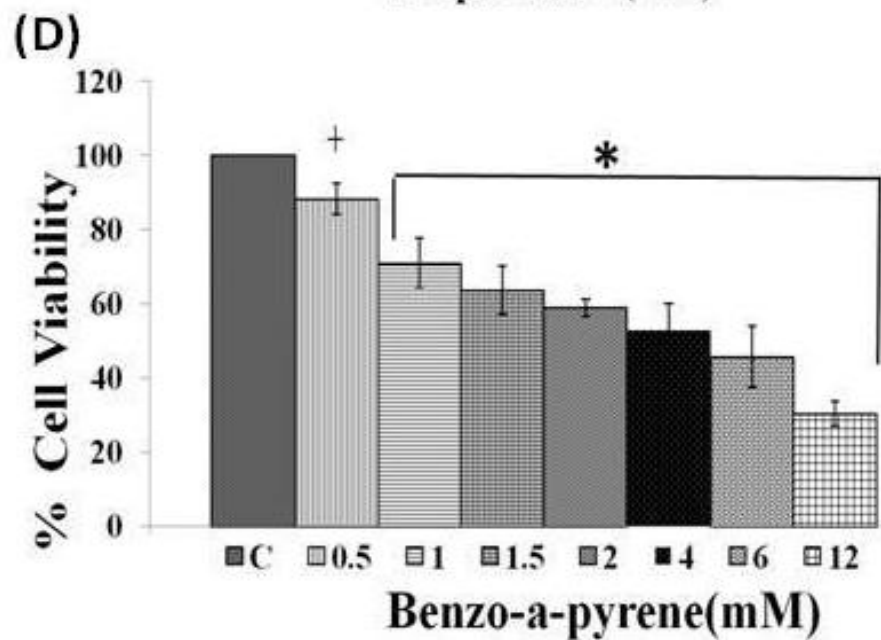

Supplement: Supplementary file 1 — Additional file 1. Figure S1: Effect of various doses of AL, STZ, DB and BP on the viability of RIN5F cells in vitro. [file 12944_2019_1164_MOESM1_ESM.pdf]

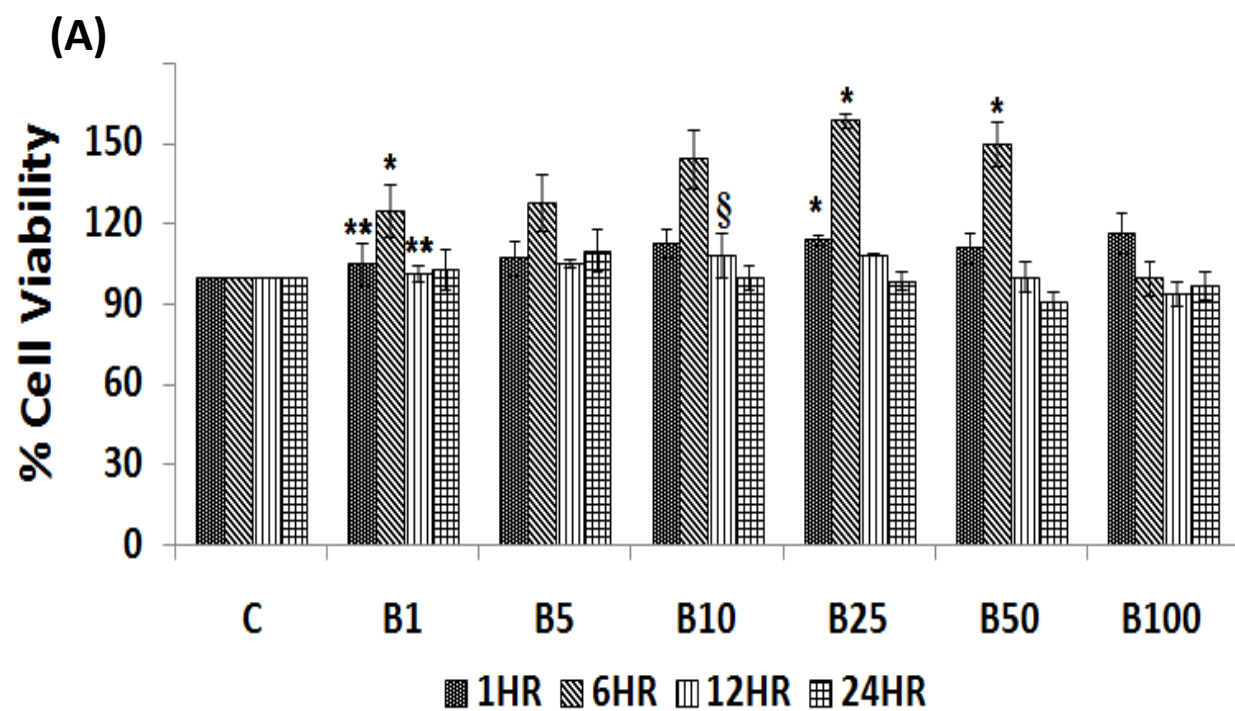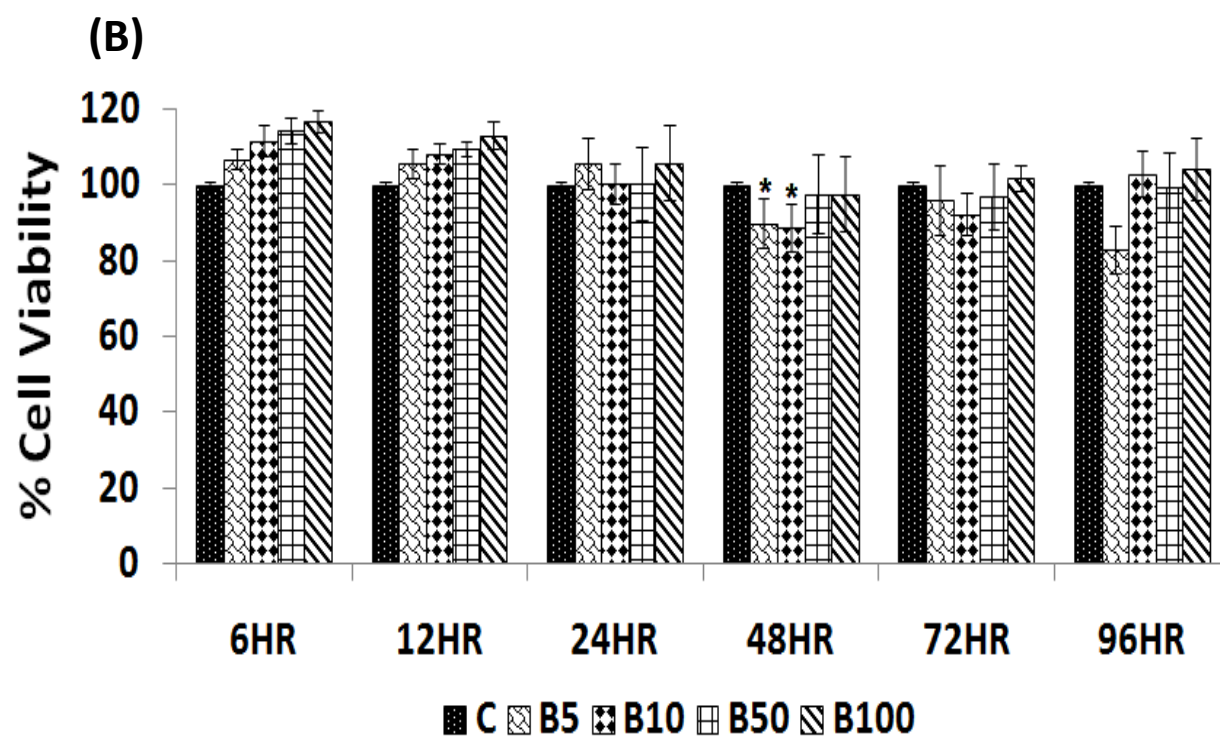

Supplement: Supplementary file 2 — Additional file 2. Figure S2: Effect of various dsoes of BDNF on the viabiltiy of RIN5F cells in vitro. [file 12944_2019_1164_MOESM2_ESM.pdf]

(A)

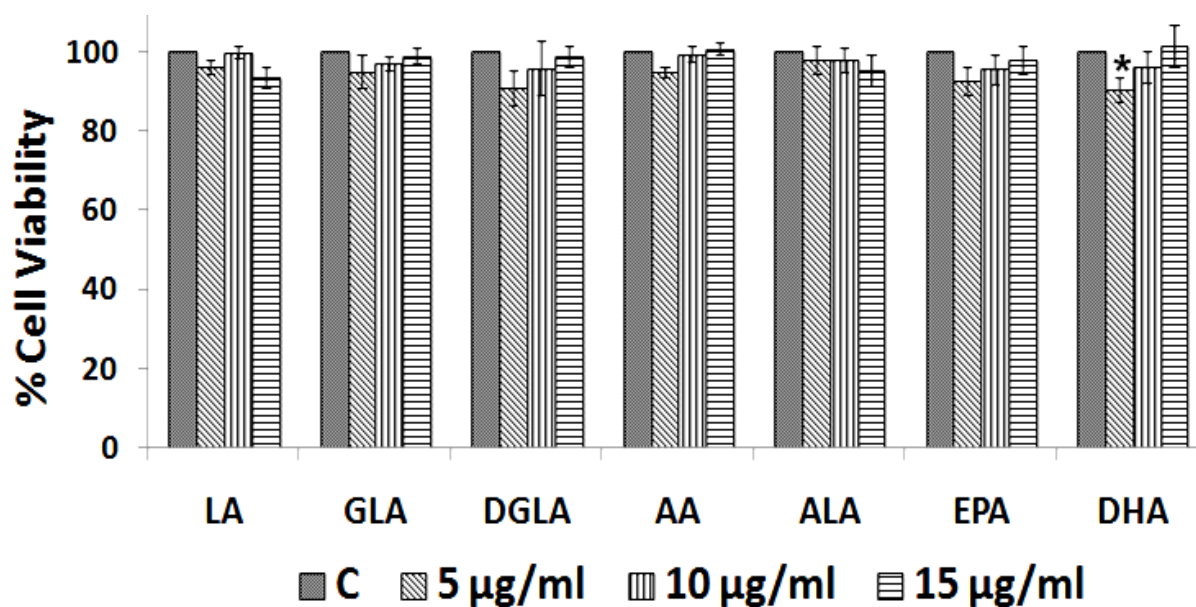

(B)

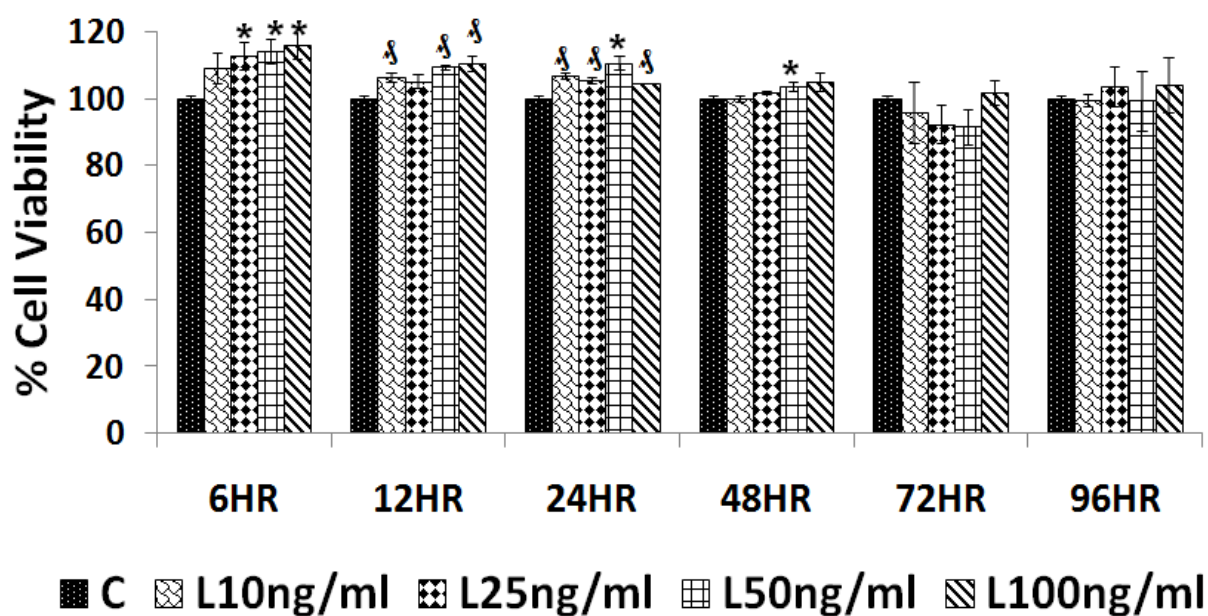

Supplement: Supplementary file 3 — Additional file 3. Figure S3: Effect of various doses of PUFAs and LXA4 on the viability of RIN5F cells in vitro. [file 12944_2019_1164_MOESM3_ESM.pdf]
